# Obesity supersizes macrophage and neutrophil activation after stroke while lipid droplets play a protective role

**DOI:** 10.1186/s12974-026-03774-7

**Published:** 2026-03-19

**Authors:** Karen Bradshaw, John Holsten, Oliver Hahn, Aulden Foltz, Kristy A. Zera, Li Zhu, Christine Haarslev, Tony Wyss-Coray, Todd C. Peterson, Marion S. Buckwalter

**Affiliations:** 1 Department of Neurology and Neurological Sciences, Stanford University School of Medicine, 1201 Welch Rd, Stanford, CA 94305-5489 USA; 2 Department of Psychology, University of North Carolina Wilmington, Wilmington, NC USA; 3 Department of Neurobiology Research, Institute of Molecular Medicine, University of Southern Denmark, Odense, Denmark; 4 VA Palo Alto Health Care System, Palo Alto, CA USA; 5 Department of Neurosurgery, Stanford University School of Medicine, Stanford, CA USA

**Keywords:** Obesity, scRNAseq, Stroke, Interferon signaling, Oxidative stress, Complement, Lipid droplet, Coagulation, Blood, Brain

## Abstract

Obesity is a major stroke risk factor. By 72 h after ischemia, obese mice exhibit greater stroke sizes, neuroinflammation, and motor deficits. However, the immune mechanisms driving these outcomes remain incompletely understood. To define how obesity reshapes the immune response to stroke, we performed single-cell RNA sequencing of immune cells in the blood and brain 72 h after stroke or sham surgery in control-diet and diet-induced obese mice. In the blood, macrophages and neutrophils from obese mice exhibited the most pronounced transcriptional changes under both sham and stroke conditions. These changes included *Ly6c2-Cd68-Cx3cr1-*enriched monocyte-derived transitioning macrophages and classic macrophage populations enriched in the lipid droplet gene perilipin-2 (*Plin2*) with one also enriched with foamy macrophage marker *Cd36*; chemotaxis-associated neutrophil subtypes enriched in *Cxcl2* or *Cxcr2;* and interferon-signaling neutrophils. Obese mice after sham surgery exhibited minimal immune changes in the brain, but after stroke, we report substantial transcriptional changes in infiltrating monocyte-derived macrophages with high or intermediate *Plin2* expression, interferon-signaling dendritic cells, and *Cxcl2*-enriched neutrophils. The obesity-induced changes in immune cells across both blood and brain are characterized by elevated lipid handling, inflammation, cellular stress, and notable coagulation pathways in the brain neutrophils. Co-expression analyses revealed that elevated *Plin2*, a hallmark of obesity, is positively correlated with lipid-related, immune, stress, and pro-thrombotic genes. We tested its role by stereotactically injecting *Plin2* siRNA into the stroke. In non-obese mice, *Plin2* knockdown increased infarct size and worsened motor outcomes despite reducing inflammatory markers (CD68, IFITM3, and TSPO), suggesting that early lipid droplet accumulation is neuroprotective and stimulates the immune response to stroke. In obese mice, *Plin2* knockdown mildly worsened motor deficits while reducing obesity-elevated CD68. Collectively, these findings identify elevated lipid handling, neuroinflammation, cellular stress, and coagulation as key transcriptional features of obesity-exacerbated stroke outcomes while revealing a protective role for *Plin2.*

**Supplementary Information:**

The online version contains supplementary material available at 10.1186/s12974-026-03774-7.

## Background

Obesity and stroke are intimately intertwined. Obesity is a major public health concern and a well-established risk factor for stroke, contributing to both its incidence and severity. It affects 43% of U.S. adults and has more than doubled in global prevalence since 1990 [1, 2]. Simultaneously, stroke is a leading cause of long-term disability, affecting more than 795,000 Americans each year. Obesity is itself a key risk factor for stroke, increasing risk by 64% compared to individuals of normal weight, while overweight individuals face a 22% higher stroke risk [3].

Obesity is thought to promote stroke and other cardiovascular diseases by driving systemic metabolic dysfunction and chronic low-grade inflammation in peripheral tissues, including blood, adipose tissue, and liver. Common pathways that are elevated in humans in these tissues are interferon alpha and gamma [4, 5], IL-6, tumor necrosis factor (TNF; [6, 7]), and alternative and classical complement pathways [8–10]. Mouse models of diet-induced obesity mirror findings in humans [4, 11–14], and prior studies have demonstrated that interferon and complement signaling can promote insulin resistance and lipid droplet accumulation [12, 15–17, 8, 9, 18–20].

In addition to increasing stroke risk, obesity also worsens stroke outcomes. After stroke, obese mice exhibit heightened neuroinflammation characterized by microgliosis [21–23], astrogliosis, increased cytokine levels [24], and peripheral immune cell infiltration [22, 23, 25–31]. This enhanced neuroinflammation is accompanied by oxidative stress and endothelial cell dysfunction which contribute to increased blood-brain barrier permeability [29, 32, 33]. Obesity also promotes neuronal apoptosis [32, 34], larger infarct sizes [22], and impaired blood flow [32, 33]. Consistent with these pathological changes, obese rodents exhibit poorer functional recovery after stroke [23, 28, 32].

In our prior work, we demonstrated that in mice with diet-induced obesity, stroke causes exacerbated neuroinflammation and stroke size expansion within 3 days compared to mice fed a normal diet [21]. We also found little change in neuroinflammation or gene transcription in the cortex with obesity in mice that did not have surgery. Bulk transcriptional analysis of the brain before and after stroke demonstrated that obesity elevates genes involved in interferon signaling, antigen presentation, and general immunity. This suggests that immune responses are amplified or made more toxic by obesity and that this contributes to early stroke expansion. However, this work did not identify which immune cell types are contributing to this response or identify their specific gene changes.

We thus aimed here to characterize the immune landscape at a single cell level in both the blood and brain of mice with diet-induced obesity with and without stroke and compare this to non-obese mice. We utilized single-cell RNA sequencing (scRNA-seq) of immune cells from the blood and brain of obese and non-obese mice, three days after sham or distal middle cerebral artery occlusion (dMCAO) surgery. This model was selected to capture ischemic stroke during the subacute phase, when inflammation peaks. We analyzed which immune cell subtypes in the blood and brain exhibit the greatest numbers of differentially expressed genes after stroke and performed pathway analysis to identify genes involved in processes exacerbated by stroke. We also performed co-expression analysis with *Plin2* to understand how obesity-affected processes interact. To complement the transcriptomic data analysis, we used immunohistochemistry (IHC) of Perilipin-2 (PLIN2), a lipid droplet marker and CD68 to characterize phenotypic changes in macrophages after stroke. In response to observing a robust obesity-induced elevation of *Plin2* expression in neutrophils and macrophages, we performed intracortical *Plin2* siRNA administration to directly test the functional role of lipid droplet accumulation early after stroke. We performed IHC for stroke size, PLIN2, CD68, IFITM3, and TSPO to assess the outcome of PLIN2 knockdown on stroke size, neuroinflammation, and cellular responses associated with oxidative stress. Additionally, we performed motor behavior assays to assess functional outcomes. Together, the scRNAseq data we produce and the *Plin2* siRNA intervention allowed us to identify how key immune cells are changing with obesity in response to stroke and determine how targeting lipid droplets during the subacute phase affects outcome.

## Methods

### Mice

We purchased 11–18 week-old male C57BL/6J mice from Jackson Laboratory (#000664) and housed them under a 12-hour light/dark schedule. Animals were group housed 2–5 mice per cage and allowed to acclimate for 3 days. Mice had *ad libitum* access to water and a high fat diet (60% kcal fat/7% kcal sucrose; no. d12492; Research Diets, Inc., New Brunswick, NJ) or a normal fat diet (18% fat/7% sucrose; no. 2018SX; Harlan Laboratories, Indianapolis, IN) for 6 weeks. All experiments were performed in accordance with protocols approved by either the Stanford University or UNCW Institutional Animal Care and Use Committees (IACUC).

### Stroke surgeries

We performed the distal middle cerebral artery occlusion (dMCAO) ischemic stroke surgeries as previously described [35]. Mice were placed under anesthesia using approximately 2% isoflurane in pure oxygen and maintained at 37 °C with a temperature-controlled heating pad throughout the surgical procedure. The surgical site was disinfected with chlorhexidine (Vetoquinol #1703001412), and an incision was made along the skull. The temporalis muscle was cut to expose the middle cerebral artery (MCA) through the skull. A craniotomy was performed directly over the MCA, the meninges were carefully removed, and the MCA was permanently cauterized. After the procedure, the muscle and skin were repositioned, and the incision was sealed with surgical glue (Meridian Animal Health #1166401654). Mice after sham surgery underwent the same surgical steps, excluding the MCA cauterization. Following the surgeries, all mice received a single subcutaneous injection of cefazolin (25 mg/kg; WG Critical NDC44567-707-25) and buprenorphine SR (1 mg/kg; Wedgewood #79926-058-17) as an antibiotic and analgesia, respectively.

For the photothrombotic stroke targeting the primary motor cortex, mice were injected intraperitoneally with 100 mg/kg of the photosensitive dye Rose Bengal (no.330000 5G, Sigma) in sterile saline and placed under anesthesia using approximately 2% isoflurane in pure oxygen and maintained at 37 °C with a temperature-controlled heating pad throughout the surgical procedure. Mice were positioned into a stereotactic frame, and a Metal Halide Fiber optic light (no.56371, Edmundo Optics) focused to a 1 mm diameter with a 10x microscope objective. This was placed stereotactically directly over the right motor cortex (0.5 mm anterior, 1.5 mm lateral to Bregma) for 15 min to induce thrombus formation. After the stroke, the incision was sealed with surgical glue (Meridian Animal Health #1166401654). Following the surgeries, all mice received a single subcutaneous injection of cefazolin (25 mg/kg; WG Critical NDC44567-707-25) and buprenorphine SR (1 mg/kg; Wedgewood #79926-058-17) as an antibiotic and analgesia, respectively. Mice recovered for 30 min on a heating pad.

### Single cell RNA sequencing

Three mice per group were anesthetized with 10 µl/g ketamine/xylazine solution (0.6 mL ketamine at 100 mg/uL, 0.8 mL xylazine at 100 mg/mL, 3.6 mL 0.9% NaCl) and perfused with 15 mL ice cold phosphate-buffered saline (PBS) following 125 uL cardiac blood collection. The mouse blood and brain tissues within each condition were pooled into a single sample for processing.

### PBMC collection

One hundred twenty-five µL of blood from each animal (*n* = 3 per group) was pooled into a single EDTA-coated tube (BD Microtainer #365974). Samples were incubated in 1250 µL of ACK buffer (Gibco, #A1049201) for 3–5 min at room temperature. The reaction was stopped with 25 mL of molecular grade 1X PBS, and cells were centrifuged at room temperature (300 g for 5 min). Supernatant was aspirated, and another 1 mL of ACK buffer was added for a 3-minute incubation at room temperature. 6 mL of PBS was added for washing, and cells were centrifuged (300 g at 4 °C for 5 min). After aspiration of PBS, cell pellets were resuspended in FACS buffer (1% BSA in PBS with 2 mM EDTA) and blocked for 5 min on ice with Fc block (CD16/CD32, BD 553141), followed by staining for 10 min with the anti-CD45-PE/Cy7 (1:200, BD 103114) antibody. Two milliliters of PBS was added to each sample before centrifugation (300 g at 4 °C for 5 min). Supernatant was removed, and 200 µL of FACsort resuspension buffer (20% ultrapure BSA in PBS with 0.5% RNAse inhibitor; Thermo Fisher #N2115) was added to the cells before they were filtered through a 100 µM strainer. Dead cells were excluded by staining with SYTOX Blue Dead Cell Stain (1:5,000, Thermo Fisher Scientific, S34857).

### Brain dissociation

Brain tissue was dissociated with the Miltenyi neural dissociation kit (Miltenyi, 130–092–628) following the manufacturer’s instructions, with minor adjustments to optimize the isolation of immune cells. The stroke core and peri-infarct cortex, defined as ~ 2 mm surrounding the visible lesion, were dissected and pooled by condition, minced, and digested using the neural dissociation kit (Miltenyi, 130–092–628). A similar cortical region was dissected from mice who underwent sham surgery. Briefly, brains were removed following saline perfusion, then cortical tissue was mechanically dissociated in HBSS and centrifuged. After the supernatant was aspirated, we added 1 mL of Enzyme P per sample at 37 °C for 10 min, followed by the addition of 30 uL Enzyme A and Enzyme Y mixture per sample with a second incubation at 37 °C for 10 min, with intermittent gentle agitation. Enzyme P, A, and Y are proprietary reagents supplied in the Miltenyi kit. Gentle mechanical trituration was performed before and between enzymatic digestion steps to facilitate dissociation while minimizing cell damage. After incubation, tissues were washed with 10 mL DPBS and passed through a 0.2 μm strainer before centrifugation. The supernatant was removed. The filtered homogenate was then centrifuged at 300 g for 10 min, and the resulting pellets were resuspended in 0.9 M sucrose in dPBS. This suspension was centrifuged again for 20 min at 850 g at 4 °C to facilitate myelin separation. The cell pellets were then resuspended in FACS buffer (1% BSA in PBS with 2 mM EDTA) and incubated on ice for 5 min with Fc block (CD16/CD32, BD 553141) to prevent nonspecific binding. Cells were stained for 10 min with anti-CD45-PE/Cy7 antibodies (1:200, BD 103114). Dead cells were excluded by staining with SYTOX Blue Dead Cell Stain (1:5,000, Thermo Fisher Scientific, S34857).

### Fluorescence Activated Cell sorting (FACs)

Peripheral blood mononuclear cells (PBMCs) and brain immune cells were sorted using the FACSAria 3.3 system (BD Biosciences) and analyzed with FACSDiva software (BD Biosciences). Cell populations were distinguished based on forward scatter (FSC) for size and side scatter (SSC) for internal complexity. FSC-A and FSC-W gating was employed to separate individual cells from doublets and aggregates. SYTOX Blue+ dead cells were also excluded via gating. The immune cell population was defined as CD45 + cells. Sorted cells were collected into the FACsort resuspension buffer, centrifuged at 1,000 r.p.m. for 10 min at 4 °C, and then resuspended in 50 µL of the FACsort resuspension buffer before being processed for single-cell RNA sequencing. Cell collection was capped at 60,000 cells per sample with all PBMC samples reaching this cap except for the obese stroke sample (56,750 cells). In the brain, all samples reached this cap except for 12,896 cells collected in non-obese sham and 10,752 collected in the obese sham samples (Supplementary Table 1).

### RNA extraction, library preparation and RNA sequencing

Reagents from the Chromium Single Cell 3’ GEM & Gel Bead Kit v3.1 (10X Genomics, 1000121) were thawed and prepared according to the manufacturer’s instructions. The cell and master mix solution was calibrated to target 10,000 cells per sample for the 10x reaction, which were then loaded onto a standard Chromium Controller (10X Genomics, 1000204) as per the provided guidelines. Resuspended samples were then re-quantified and loaded as per the manufacturer instruction. A total of 11 PCR cycles were performed to synthesize cDNA. Library construction was carried out using the Chromium Single Cell 3’ Library Construction Kit v3 (10X Genomics, 1000121), following all reaction and quality control procedures as specified by the manufacturer with the recommended reagents, consumables, and equipment. Quality assessments of cDNA and libraries were performed using a Bioanalyzer (Agilent) at the Stanford Protein and Nucleic Acid Facility. Sequencing of the prepared libraries was conducted by Novogene on an Illumina NovaSeq S4 platform (Illumina), with base calling, demultiplexing, and FastQ file generation handled by Novogene.

### Data processing and analysis

Fastq and metadata files were uploaded to NCBI (#GSE310324). The Cell Ranger (v.6.0.0) analysis pipeline was employed to align sequencing reads to the mm10-reference genome and quantify barcodes and unique molecular identifiers (UMIs). Reads mapping to introns were excluded. Cells with a high mitochondrial content (> 20%) relative to endogenous RNAs were removed in Seurat. Cells were also removed if they expressed fewer than 100 unique genes. After filtering, the following cell counts remained in blood; 7,486 (control diet sham), 5,722 (control diet stroke), 7,519 (high-fat diet sham), and 6,710 (high-fat diet stroke). In the brain, final cell counts were 5,477 (control diet sham), 11,068 (control diet stroke), 5,236 (high-fat diet sham), and 12,026 (Additional File 1- Supplementary Table 1; high-fat diet stroke). Genes not detected in any cell were removed from subsequent analysis. This method achieved an average of over 5,000 reads per cell.

Log-normalized expression values (scale factor: 10,000, pseudocount: 1) were generated using Seurat’s NormalizeData function and used for clustering and differential gene expression analysis. Principal component analysis (PCA) was conducted on the 2,000 most variable genes, and UMAP was performed on the first 20 principal components using the FindNeighbor function (we used the default k-nearest neighbor parameter, k.param = 20) in the Seurat R package (v4.3.0.1). Clustering was performed with the FindClusters()algorithm using a resolution parameter of 0.4 for PBMCs and 0.6 for the brain cells. Cell types were identified using marker genes generated by the Seurat function FindAllMarkers() (Additional File 2- Supplementary Table 2). Marker genes were identified using Seurat’s FindAllMarkers function with the Wilcoxon rank-sum test, applying a minimum detection threshold of 25% of cells per cluster (min.pct = 0.25). Genes were considered significant if they met a Bonferroni-corrected adjusted p-value < 0.05, as implemented by default in Seurat. Cell types were identified manually by referencing the CellMarker 2.0 database and the primary literature.

From the PBMC samples, one cluster identified as contaminating red blood cells was removed. From the brain dataset, one contaminating red blood cell cluster, one mixed endothelial/astrocyte/neuron cluster, and one unidentified brain cluster were removed. Doublets were identified using DoubletFinder (v2.0.6) applied per Seurat cluster (PCs 1–10, pN = 0.25, and pK = 0.1, expected doublet rate set to 7.5% of cells). Removal of predicted doublets did not alter differential expression results in the most affected cell types (ranked by the number of obesity-induced differentially expressed genes following stroke, Additional File-Supplementary Fig. 1). All cells were thus retained for primary analyses to preserve statistical power and potential rarer transitional immune cells. UMAP visualizations by condition and clusters were generated using ggplot2 (v3.4.4). Cell counts were calculated after subsetting clusters by condition. Differential gene expression analyses between stroke, sham, obese, and non-obese conditions were performed using Mast (v. 1.24.1). Only genes expressed by at least 10% of cells in at least one condition of each pairwise comparison were included. Log-normalized RNA-counts were used for differential gene expression analysis. Overrepresentation analysis was performed with Enrichr [22–24]. This analysis includes differentially expressed genes (fdr cutoff: 0.05). Identified pathways are from the MSigDB Hallmark 2020 database.

### *Plin2* expression and gene correlation analysis

Normalized gene expression was extracted from the RNA assay of the full Seurat object after restricting cell subtypes with detectable *Plin2* expression. If detected, the expression vector of *Plin2* was extracted and pairwise correlations with every other gene in the dataset were computed using Spearman’s rank correlation coefficient. This approach was selected because it does not assume linear relationships and is robust to outliers, making it suitable for scRNA-seq data.

### Fluorescent immunohistochemistry

Mice were perfused with saline and brain tissue was fixed in 4% PFA overnight and then placed in 30% sucrose. Cryosections (40 μm thick) were processed using a free-floating immunofluorescence protocol in mesh inserts within 24-well plates. Sections were washed in TBS buffer, then blocked for 1 h at room temperature using 3% goat serum and 0.3% Triton X-100 in TBS. Primary antibodies against Perilipin-2 (Santa Cruz #sc-44842) and CD68 (Bio-Rad/AbD Serotec, #NC9471873) were diluted in 1% goat serum with 0.3% Triton X-100 and incubated overnight at 4 °C. Following TBS washes, appropriate Alexa Fluor-conjugated secondary antibodies at 1:200 (goat anti-rabbit 488, anti-rat 555, and anti-chicken 647) were applied for 1–2 h at room temperature in the dark. After final washes, sections were mounted on Superfrost slides with Vectashield mounting medium, with DAPI, and stored at 4 °C. Negative and positive controls were included to assess staining specificity and background fluorescence. For oil red staining, free-floating sections were washed three times with phosphate-buffered saline (PBS). Slides were then incubated in absolute propylene glycol for 5 min to prevent water carryover into the Oil Red O solution. Oil Red O solution, pre-warmed to 55 °C, was applied to the section for 15–20 min. Following staining, slides were rinsed in 85% propylene glycol for 5 min to remove excess dye and subsequently washed three times with PBS. Slides were then washed three times with PBS. After final washes, sections were mounted on Superfrost slides with Vectashield mounting medium, with DAPI, and stored at 4 °C.

Fluorescent images were taken on a SP8 confocal microscope (Lecia) using a 40X oil immersion objective in two regions of interest (dorsal and ventral) within the core. Images were composited and intensity of fluorescence were analyzed by a blinded scientist using ImageJ after setting a consistent threshold value across all tissues.

### DAB immunohistochemistry

Staining of 3,3’-diaminobenzidine (DAB) was performed on PFA-fixed 40 μm coronal brain sections. Sections were incubated overnight at 4 °C in primary antibody against PLIN2, NEUN (Santa Cruz #sc-44842, Sigma-Aldrich, #12352203), IFITM3 (abcam, #ab15592), or TSPO (abcam, #ab109497). Sections were then incubated at room temperature for an hour in a secondary biotinylated antibody against the host of each primary antibody. Images for PLIN2, CD68, IFITM3, and TSPO were taken using a Keyence BZ-X710 microscope. Quantification was performed on 1–2 10x images of the stroke core per section per mouse (most dorsal and ventral), by a researcher blinded to the treatment group. Immunostains of PLIN2, CD68, IFITM3, and TSPO were quantified using thresholding in ImageJ and reported as % area of the stroke core covered by antibody staining. The specificity of primary antibody staining was confirmed using control sections treated with secondary biotinylated antibody alone. NEUN-stained sections for stroke size were imaged with a PathScan Enabler IV and the infarct and hemisphere area were traced with ImageJ software (version 1.51c, 2017). The percent of infarcted hemisphere was used to quantify area of stroke in the ipsilateral hemisphere [21].

### siRNA injections

Small interfering RNA (siRNA) against *Plin2* (Santa Cruz Biotech, #sc-44842) and scrambled control siRNA (Santa Cruz Biotech, # sc-37007) were prepared at 1 ug/uL in i*n vivo*-jetPEI (Avantor, #89129-960) according to the manufacturer’s instructions. One day after photothrombotic stroke or sham surgery, mice were placed under anesthesia and positioned on a stereotactic frame. A craniotomy was performed to reveal the primary motor cortex where the initial stroke was induced, and two 2uL injections were performed at 0.5 uL/min: the first one at 0.9 mm anterior, 1.75 mm lateral to Bregma, and the second at 1.0 mm anterior and 1.25 lateral mm to Bregma. The incision was sealed with surgical glue (Meridian Animal Health #1166401654). Mice recovered for 30 min on a heating pad.

### Motor behavior protocol

To measure functional recovery following photothrombotic stroke to the motor cortex, we utilized tapered beam and rotating rod tasks. The mice were handled for 5 days before we began to implement the motor assays. Mice were allowed to habituate to the room for 1 h before we began handling or starting the assays. For each assay, the mice underwent one training day followed by two days of 3 trials of the rotating rod and tapered beam. The second day was set as baseline performance before stroke surgery and animals were tested at 2 and 3 days after stroke with 3 trials each.

### Rotating rod

The rotating rod was used to test gross motor performance as described [21]. The mice were placed on a 140 cm rotating beam that rotates around its long axis at 6 rpm and is attached to an escape box. Distance travelled before the mice fell off the beam and speed were recorded. For the training day, the mice were first placed on the rod without the motor turned on. They were placed on this still rod until they were able to successfully cross the entire distance without falling. Once all mice completed this, the motor was turned on, and the mice were placed on the rotating rod until they were able to successfully cross the entire distance. The mice repeated this task for 3 trials per day for two days before stroke and two and three days after stroke. Mice that were alive but unable to move on the rod at all were assigned the worst score across all mice for a particular timepoint.

### Tapered beam task

The tapered beam task, as described [21], was used to test fine motor function of the front left paw, which was the primary target of the photothrombotic stroke. The tapered beam becomes more narrow from the top to the bottom and is angled at 30 degrees from the escape box. For the training day, the mice were placed at the top of the beam until they were able to cross it completely into the escape box. They then repeated this for 3 trials per mouse per day for two days before the stroke and at two and three days after stroke. The second day was set as the baseline for performance for evaluating post-stroke motor performance. The distance before the first time the left front paw slips off, the total number of left front paw slips, and the time it takes for the mice to complete travel down the tapered beam to the escape box were recorded. Mice that were alive but unable to complete a task were assigned the worst score across all mice for a particular timepoint.

### Statistical analysis

For all experiments, animals were randomized and concealed, and experimenters were blinded during all procedures, tissue imaging, and analysis. For behavior, the only animals excluded completely were those that died during the siRNA injection step. Mice that died within 2 or 3 days of stroke were excluded from behavioral analysis starting at the time of death. Mice that were alive but unable to complete a task were assigned the worst score across all mice for a particular timepoint. GraphPad Prism Software (version 8) was used for graphing and statistical analyses. For behavior, a mixed effects model was used. The model included time, treatment, and time × treatment interaction as fixed effects. Sphericity was not assumed, and the significance level (alpha) was set at 0.05. Post hoc comparisons were performed using Tukey’s multiple comparisons test to evaluate pairwise differences between treatment groups at each time point. The difference of means for immunohistochemistry was assessed by the two-tailed Student’s t test or one-way ANOVA with post hoc Kruskall-Wallis test.

## Results

### Impact of obesity and stroke on blood and brain immune cell composition

**Fig. 1:**
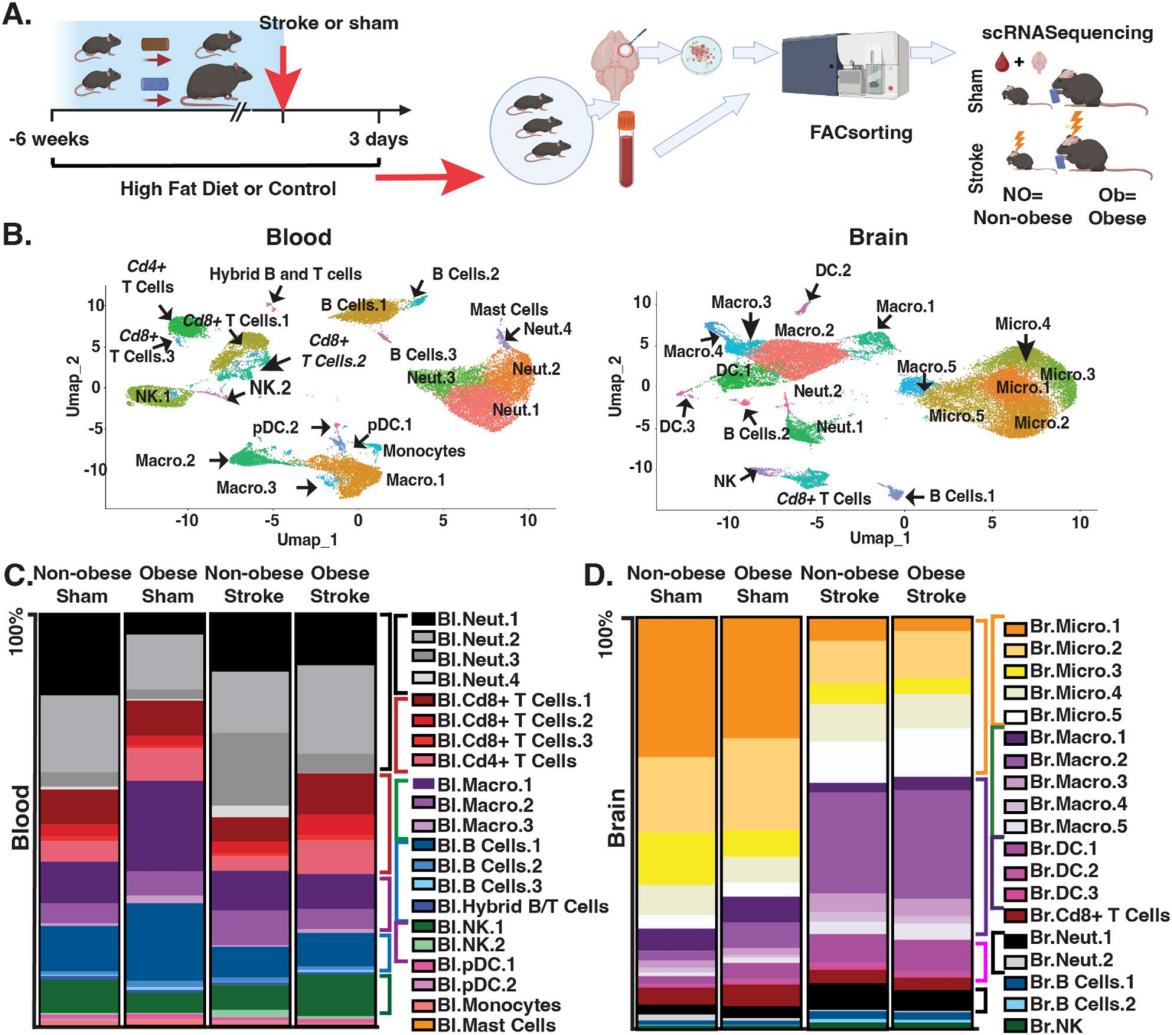
Experimental Design and Cell Type Proportions. **A** Experimental Design. Mice (*n* = 3 per group) were fed a high fat or control diet for 6 weeks followed by dMCAO stroke or sham surgery. Seventy-two hours after surgery, cortical tissue and blood samples from 3 mice per condition were pooled before FAC sorting for CD45 + cells and subsequent scRNA-Seq. NO, non-obese Ob, obese. **B** UMAPs of annotated cell types in the blood (left) and brain (right). **C **Percentage of annotated cell types in total sequenced blood population. **D** Percentage of annotated cell types in total sequenced brain cell populations. Neut, neutrophils; Macro, macrophages; NK, natural killer cells; pDC, plasma dendritic cells; Micro, microglia

To understand the impact of obesity and stroke on peripheral and central inflammation, we performed single cell RNAsequencing (scRNA-Seq) on blood and brain immune cells from four groups: non-obese and obese mice with either dMCAO or sham surgery (Fig. 1a). We selected the distal middle cerebral artery occlusion (dMCAO) model because it produces a highly reproducible infarct size and location, enabling us to more precisely isolate obesity-dependent immune responses and inflammatory mechanisms. Mice fed a high fat diet displayed significant weight gain and developed insulin resistance (Additional file 4- Supplementary Fig. 2). Mice were sacrificed 72 h after surgery and as expected, we reached a collecting capacity of 60,000 cells in stroke brain tissue but not in sham tissue (Additional File 1: Supplementary Table 1). We sequenced CD45 + cells from the blood and brain and then used Seraut to generate UMAPs (Fig. 1b). We then used reference datasets (CellMarker 2.0) and published data to assign each cluster from the UMAP a major immune cell type and subtype identity in blood (Bl) and brain (Br; Additional File 2- Supplementary Table 2), which contains the markers we used to define each cell subtype. Notably, macrophages that are enriched in both classic monocyte genes (*Ccr2* and *Ly6c2*) and differentiated macrophage markers (*Lyz2*,* Cd68*,* Cx3cr1*, and *Adgre1*) are likely transitional immune reactive macrophages that are often characterized in the literature as “monocyte-derived macrophages” [36–38].

Although samples were pooled from three mice per condition, we quantified the proportion of each cell subtype within each condition and considered a change of more than 10% to be likely important. Proportions of cell subtypes within the blood or brain are shown in Fig. 1c and d, respectively. As expected, obesity in mice after sham surgery roughly doubled the overall proportion of macrophages in the blood from 16% to 30%. Notably, this increase was predominantly due to increases in the lipid-enriched *Cd36-/Plin2* + monocyte-derived macrophage subtype Bl.Macro.1.

Stroke only mildly increased the proportion of total neutrophils in the blood of non-obese mice, from 42% to 47%. In obese mice there was a more pronounced increase from 21% to 39%. Additionally, after stroke the proportion of circulating T cells in obese mice was nearly doubled compared to non-obese mice (24% vs. 13%), qualitatively suggesting a stronger adaptive immune response in obese mice.

Overall, obesity did not substantially change cell subtype proportions in the brain regardless of stroke status. However, stroke in non-obese and obese mice qualitatively increased the proportion of lipid-enriched monocyte-derived macrophages Br.Macro.2 by 5-fold in the brain, pointing to the importance of these cells in general stroke outcomes. The population of Br.Macro.2 started at similar proportions in the non-obese and obese mice who underwent sham surgery (2% in non-obese, 6% in obese) and increased to similar levels after stroke (25% in non-obese, 26% in obese), highlighting the role of lipid-laden immune cells in the stroke response regardless of obesity status. There was also a ~ 2-fold increase in non-lipid-enriched infiltrating monocyte-derived macrophages (Br.Macro.3) in both non-obese and obese mice.

### Obesity upregulates lipid-related genes in the blood of mice that underwent sham surgery

To pinpoint where obesity most strongly impacts peripheral and central immune responses to stroke, we quantified the number of differentially expressed genes in each immune cell subtype. Detailed information about gene expression in each cell subtype, as well as comparisons between expression in all of the cell subtypes we studied is available in an easy to use online shiny app at https://buckwalterlab.shinyapps.io/scrnaseq_hfd_shiny_app/.

**Fig. 2:**
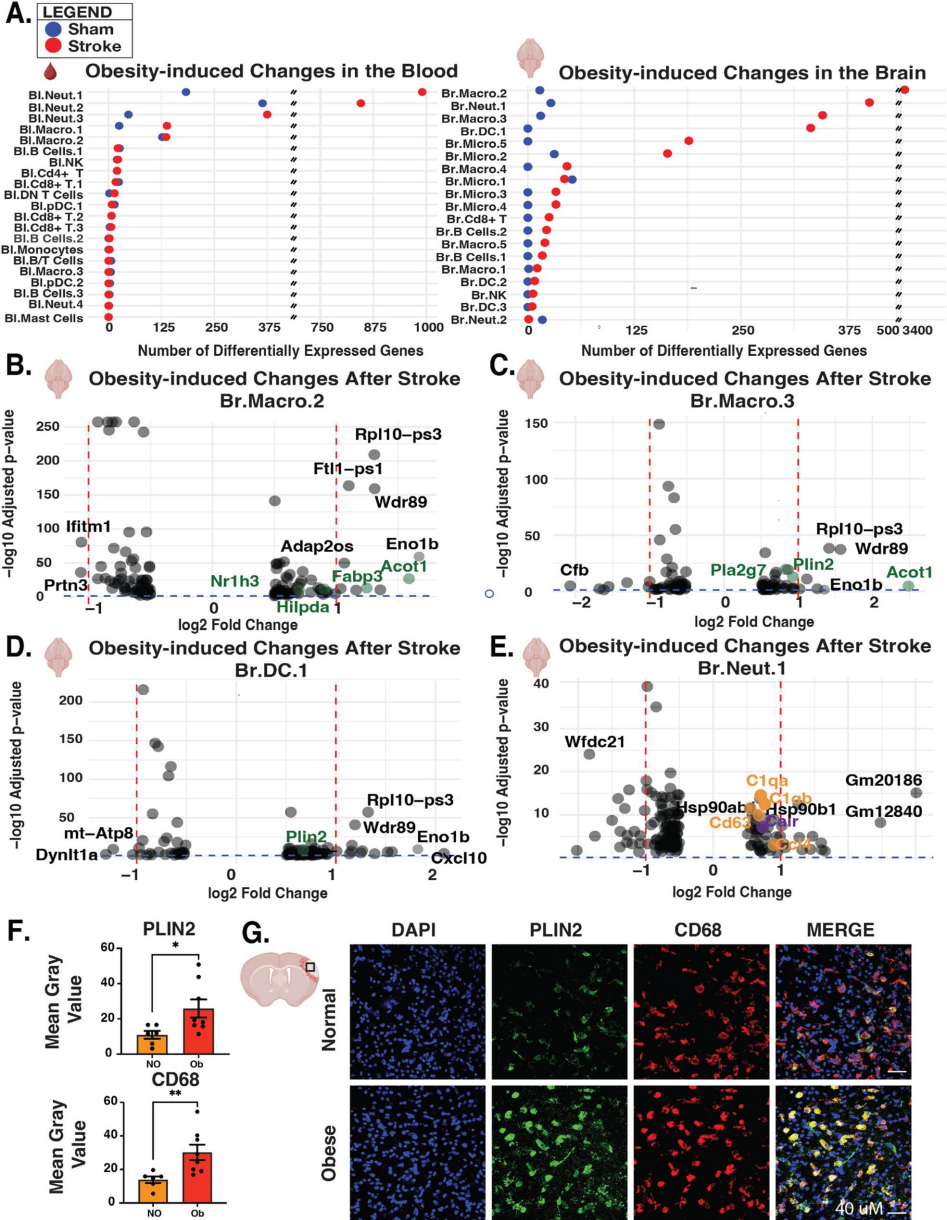
Obesity promotes lipid droplet-laden macrophages in the blood and lipids in the brain after stroke. **A** Quantification of differentially expressed genes between obese and non-obese immune cells blood (left) and brain (right). Blue circles denote the number of differentially expressed genes in obese relative to non-obese sham mice. Red circles indicate differentially expressed genes in obese relative to non-obese mice with stroke. fdr cut-off = 0.05. **B**-**E** Volcano plot of differentially expressed genes (Obese Vs. Non-obese Stroke) in Br.Macro.2 (**B**), Br.Macro.3 (**C**), Br.DC.1 (**D**), and Br.Neut.1 (**E**). Log₂FC for visual of volcano plots cut-off = |0.5| and fdr = 0.05. Lipid-handling genes are labeled in green, complement signaling genes are labeled in orange, and coagulation-related genes are labeled in purple (**F**) Quantification of mean grey value of PLIN2 (top) and CD68 (bottom) in the stroke core of non-obese (NO) and obese (Ob) mice at 18–19 weeks of age. **G** Representative images (40x objective, *n* = 6–8 animals, 2–6 sections)

To analyze differential gene expression, we first compared obese to non-obese mice that underwent sham surgery. Of the five cell subtypes that had the highest numbers of significantly changed genes in the blood, two were macrophages and three were neutrophil subtypes (Fig. 2a, left, blue circles). Both subtypes of macrophages were lipid-laden with monocyte-derived macrophages Bl.Macro.1 and classic macrophage Bl.Macro.2 enriched for *Plin2* without and with *Cd36* enrichment, respectively. The three neutrophil subtypes were two chemotaxis gene-enriched subtypes, Bl.Neut.1 and Bl.Neut.2, and the interferon signaling-enriched Bl.Neut.3. Within these five cell subtypes most changed by obesity, we examined differentially expressed genes (Additional File 4- Supplementary Fig. 1a). Lipid-related genes uniquely stood out amongst upregulated differentially expressed genes in 4 of these 5 cell subtypes, with free fatty acid-producing *Acot1* and lipid droplet accumulating gene *Plin2* increasing in all but Bl.Macro.2.

In contrast to the blood, we observed very little obesity-induced transcriptional change in the brains of mice that underwent sham surgery. Most changes occurred in microglia, with < 60 differentially expressed genes (Fig. 2a, right; blue circles).

### Obesity upregulates lipid-related genes in the blood and the brain after stroke

After stroke, obesity again most strongly affected gene transcription in lipid-enriched macrophages and chemotaxis-associated neutrophil subtypes in both the blood and in the brain (Fig. 2a). In the blood they comprised the top 5 changed cell subtypes while in the brain they comprised the top three. In contrast to after sham surgery, brain immune cells also exhibited obesity-induced transcriptional changes after stroke in most immune cell subtypes, with a dendritic cell subtype and two microglial cell types most changed.

In the blood, the neutrophil and macrophage cell subtypes affected by obesity displayed even more differentially expressed genes after stroke, most notably in the *Cd36-/Plin2* enriched Bl.Macro.1 (Fig. 2a, left; red circles). In the brain, obesity triggered substantial transcriptional responses in two infiltrating monocyte-derived macrophage subtypes (Br.Macro.2 and Br.Macro.3, both *Ly6c2*-enriched and with high and medium *Plin2* expression, respectively) and a chemotaxis-associated neutrophil subtype (Br.Neut.1, Fig. 2a, right; red circles), three cell subtypes that resemble the obesity-responsive populations in the blood. In addition to macrophages and neutrophils, the subtype with the fourth highest number of differentially expressed genes in the brain was an interferon signaling dendritic cell subtype, Br.DC.1. The next two with over 125 changed genes were microglial subtypes, the complement-enriched Br.Micro.5 and the *Ccl3* and *Ccl4*-enriched chemokine-expressing Br.Micro.2. All remaining cell subtypes had fewer than 60 obesity-changed genes.

We next examined the genes upregulated by obesity after stroke, and again lipid-related genes were among the most affected in key immune populations across the blood and brain. In the blood, obesity consistently upregulated the lipid droplet gene (*Plin2)*, free fatty acid production *(Acot1*) and triglyceride synthesis genes (*Dgat1*,* Lpin2*) in macrophages and neutrophils, indicating a stroke-independent process of free fatty acid production and sequestration, most pronounced in the lipid-enriched macrophages (Additional File 5- Supplementary Fig. 3). Likewise, in the brain, obesity upregulated *Plin2*,* Acot1*,* Fabp3*, and *Pla2g7 in* the macrophage subtypes Br.Macro.2 and Br.Macro.3 (Fig. 2b and c). Br.DC.1 displayed modest *Plin2* induction along with robustly elevated *Eno1b* (Fig. 2d), while interestingly Br. Neut.1 diverged and did not strongly upregulate lipid-related genes but did strongly upregulate complement signaling genes (Fig. 2e).

To ask if the transcriptional elevation of lipid-related genes was reflected at the protein level, we next examined PLIN2 and CD68 protein as markers of lipid droplet accumulation and macrophage phagocytic activity that would engulf lipids, respectively. In the stroke core, most of the macrophages that were immunostained for CD68 were also expressing PLIN2. Obesity approximately doubled the intensity of both proteins compared to non-obese mice after stroke (Fig. 2f, g). This is consistent with the doubling of the average gene expression of *Plin2* in Br.Macro.2&3 in obese compared to non-obese mice after stroke (Additional File 6- Supplementary Fig. 4). We also see elevated oil red, a neutral lipid marker, in the stroke core of obese mice (Additional File 7-Supplementary Fig. 5:).

### Obesity enhances interferon signaling and disrupts protein stability pathways in lipid-enriched monocytic cells after stroke

To further explore how obesity alters the immune response to stroke, we performed an overrepresentation pathway analysis on differentially expressed genes in key immune cell subtypes–macrophages, neutrophils, and dendritic cells–in obese compared to non-obese mice with stroke. We focused on these most transcriptionally dynamic subtypes, comparing log_2_fold changes of representative genes between obese and non-obese mice that underwent either stroke or sham surgery, to assess how obesity modulates the magnitude and direction of the immune response after stroke in the blood and in the brain. Bl.Macro.1 and Bl.Macro.2 were the most changed monocytic cells in the blood. From the brain, Br.Macro.2 was the most changed cell subtype along with Br.Macro.3 and interferon signaling-enriched dendritic cells, Br.DC.1.

**Fig. 3:**
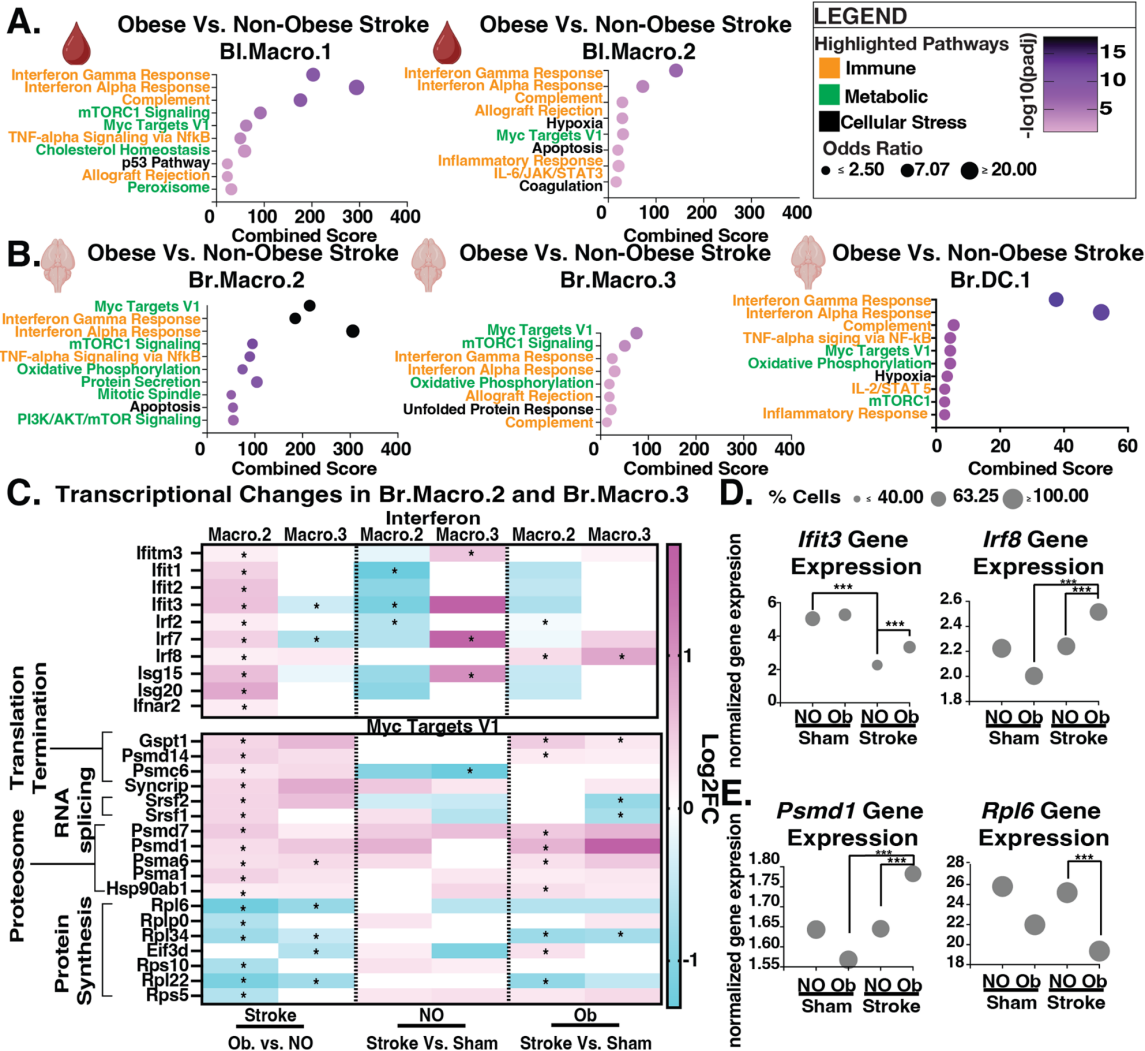
Obesity dampens interferon signaling regulation and protein stability in macrophages and dendritic cells after stroke. **A**-**B** Top obesity-altered pathways from MSigDB Hallmark 2020. Genes include all up and down-regulated genes with a cut-off of fdr < 0.05, **A** Blood macrophages Bl.Macro.1 (left) and Bl.Macro.2 (right). **B** Brain macrophages Br.Macro.2 (left), Br.Macro.3 (middle), and dendritic cells Br.DC.1 (right). **C** Heatmap of select interferon and Myc Targets V1 genes in brain macrophages comparing log₂ fold change (log₂FC) between obese (Ob) and non-obese (NO) mice after stroke (left column), and stroke vs. sham within each group (right two columns). *FDR < 0.05. **D**-**E** Normalized expression of representative (**D**) interferon (*Ifit3*,* Irf8*) and **E** Myc Targets V1 (*Psmd1*,* Rpl6*) genes in Br.Macro.2. Dot size reflects the percentage of cells expressing each gene

In obese mice with stroke, both blood macrophage cell types showed activation of interferon-alpha and -gamma signaling and the cell growth and proliferation pathways mTORC1 and downstream C Myc Target V1 (Figs. 3a). Interestingly, some of these changes were already induced by obesity in mice with sham surgery, particularly C Myc Target V1 in Bl.Macro.1, which was highly upregulated by obesity (Additional File 8-Supplementary Fig. 6. left). In Bl.Macro.2 there was strong obesity-induced upregulation of interferon alpha and gamma pathways, also mimicking the changes seen after stroke (Additional File 8- Supplementary Fig. 6. right).

Interferon signaling is indeed an established immune component in human [4] and rodent obesity [4, 11–13, 16], and in macrophage subtypes from mice with stroke, interferon signaling was highly increased by obesity in the most expanded cell type, the lipid-enriched high *Plin2*-expressing Br.Macro.2 (Fig. 3b). Interestingly, this seems to be due to a lack of downregulation after stroke in obese mice (Fig. 3c). For example, non-obese mice with stroke significantly downregulated Type I/II Interferon responsive genes such as *Ifit3* in Br.Macro.2, displaying a suppression of inflammation (Fig. 3d). In contrast, Br.Macro.2 cells from brains of obese mice didn’t exhibit as much overall downregulation of this and other interferon pathway genes, and the interferon-γ responsive gene *Irf8* was even upregulated, resulting in a stronger interferon response in Br.Macro.2 cells after stroke. Obesity also promoted interferon signaling in the interferon gene-enriched dendritic cell population Br.DC.1 from obese mice (Fig. 3b). In contrast, Br.Macro.3, a subtype with medium *Plin2* expression, exhibited obesity-induced increases in both interferon pathways that were not as robust as those in Br.Macro.2.

Another strongly enriched pathway in many macrophage subtypes of obese mice was the C Myc Targets VI pathway, important for cell proliferation and survival. This pathway was the top changed pathway in Br.Macro.2 cells and examining individual gene changes revealed disrupted protein homeostasis in obese mice. Genes involved in protein degradation were elevated and protein synthesis genes reduced in Br.Macro.2 in obese compared to non-obese mice after stroke (Fig. 3c). For example, while the macrophages of non-obese mice showed minimal transcriptional changes within this pathway after stroke, obese mice exhibited increased expression of proteasome genes like *Psmd* and reduced expression of protein synthesis genes such as *Rpl6* 1 in the macrophages (Fig. 3c and e).

### Obesity amplifies inflammatory, oxidative stress, and pro-thrombotic pathways in brain neutrophils after stroke

**Fig. 4:**
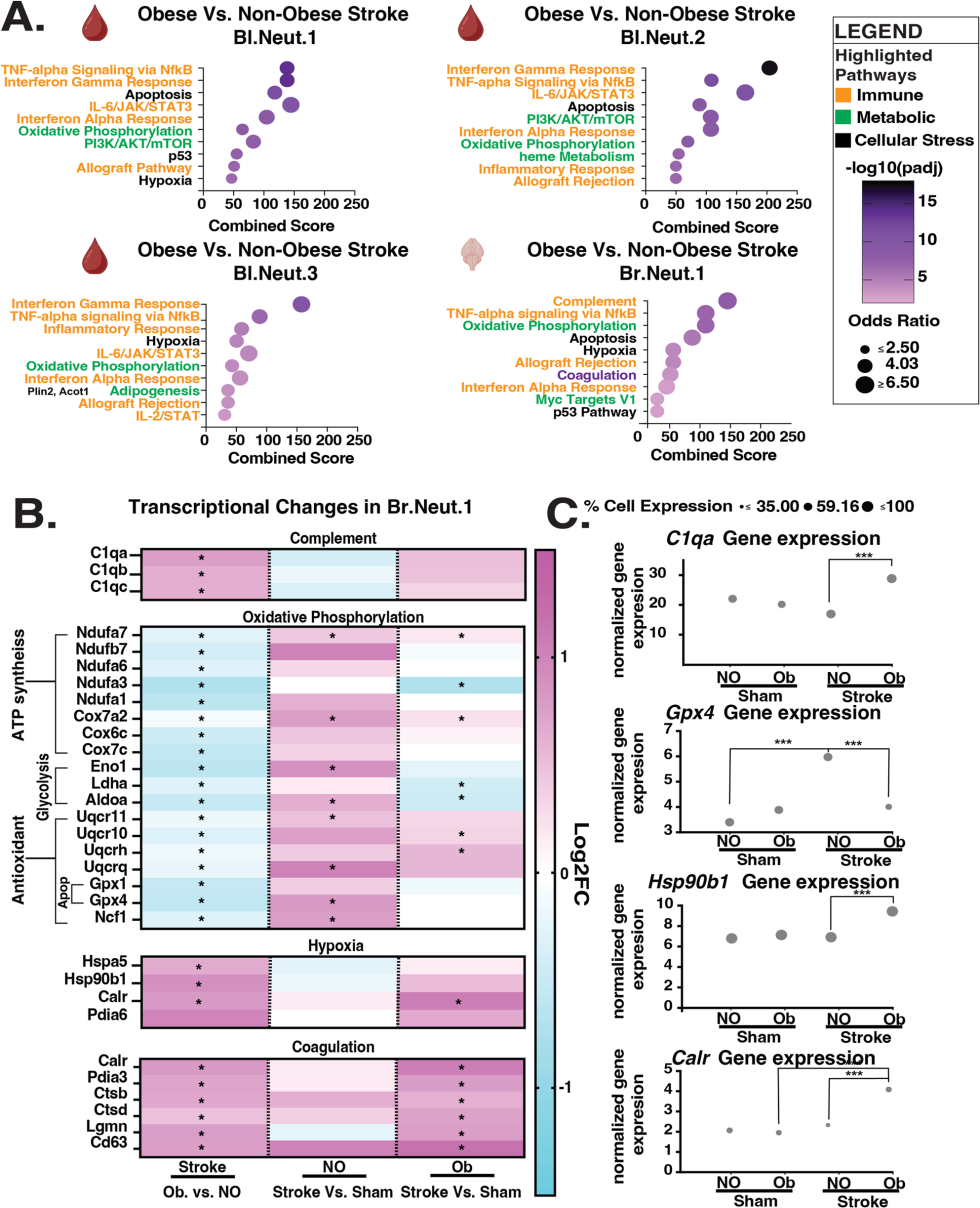
Obesity polarizes neutrophils to a pro-inflammatory state and incapacitates cellular stress response after stroke. **A** Top 10 obesity-enriched pathways in Bl.Neut.1, Bl.Neut.2, Bl.Neut.3, and Br.Neut.1 based on MSigDB Hallmark 2020 analysis (FDR < 0.05). **B **Heatmap showing log₂ fold changes in select genes from Complement, Oxidative Phosphorylation, Hypoxia, and Coagulation pathways (Apop, Apoptosis). Columns represent Obese vs. Non-obese mice after stroke, and Stroke vs. Sham within each group. *FDR < 0.05. **C** Normalized expression (scaled counts and percent of cells expressing) of representative genes: *C1qa* (Complement), *Gpx4* (Antioxidant), *Hsp90b*1 (Hypoxia), and *Calr* (Coagulation). Dot size indicates percentage of expressing cells

We next examined gene pathway changes in blood and brain neutrophils. In the blood, overrepresentation analysis highlighted pathways consistent with lipid, interferon, and TNF signaling in comparing obese to non-obese mice after sham surgery (Additional File 8-Supplementary Fig. 6.a). In the brain, there were some similarities to blood neutrophils in terms of obesity-induced genes after stroke. These included enrichment of TNF signaling via NFκB, interferon signaling, oxidative phosphorylation, and hypoxia genes. However, the brain neutrophils diverged in that complement signaling was the top affected pathway in Br.Neut.1 but was not a top pathway in any blood neutrophil subtype (Fig. 4a). Also, while non-obese mice showed little change in the transcription of classical complement activators (*C1qa*, *C1qb*, *C1qc*) in response to stroke, Br.Neut.1 from obese mice upregulated these genes after stroke, pointing to an obesity-specific activation of complement signaling in the context of stroke (Fig. 4b and c).

In addition, many genes in the oxidative phosphorylation and hypoxia pathways, both related to cellular stress responses after stroke, were polarized in brain neutrophils from obese vs. non-obese mice. While neutrophils in non-obese mice significantly upregulated mitochondrial and oxidative stress-handling genes related to ATP production and antioxidant defense after stroke, obesity drove a downregulation or dampened response (Fig. 4b). For example, *Gpx4*, an antioxidant that protects against lipid peroxidation under stress and effectively suppresses apoptosis and ferroptosis in cancer and diabetes [39–43], was upregulated after stroke in Br.Neutr.1 of non-obese but not obese mice after stroke, implying that obesity causes vulnerability to oxidative damage and consequential apoptosis in the obese group (Fig. 4b and c). At the same time, non-obese mice do not upregulate the expression of hypoxia-responsive genes such as *Hsp90b1* like Br.Neut.1 do in obese mice, suggesting that obesity promotes cellular stress that induces a heat-shock response.

Br.Neut.1 in obese mice also exhibited changes in a coagulation pathway that were due to elevated expression of coagulation genes compared to non-obese mice. This aligns with changes in blood neutrophils, where the classic coagulation marker *F5* and NETosis marker *Padi4* are upregulated by obesity (Additional File 7- Fig. 4, Additional File 8- Supplementary Fig. 6.b). Similarly in the brain, genes involved in platelet activation (*Cd63*, *Lgmn*), extracellular matrix degradation (*Ctsb*, *Ctsd*), and thrombosis (*Calr)*, were not significantly altered in brain neutrophils by stroke in non-obese mice but were upregulated in obese mice *(*Fig. 4b and c). This illustrates an obesity-driven shift toward a pro-thrombotic transcriptional profile.

### *Plin2 *is positively correlated with other pathways enhanced by obesity after stroke

**Fig. 5:**
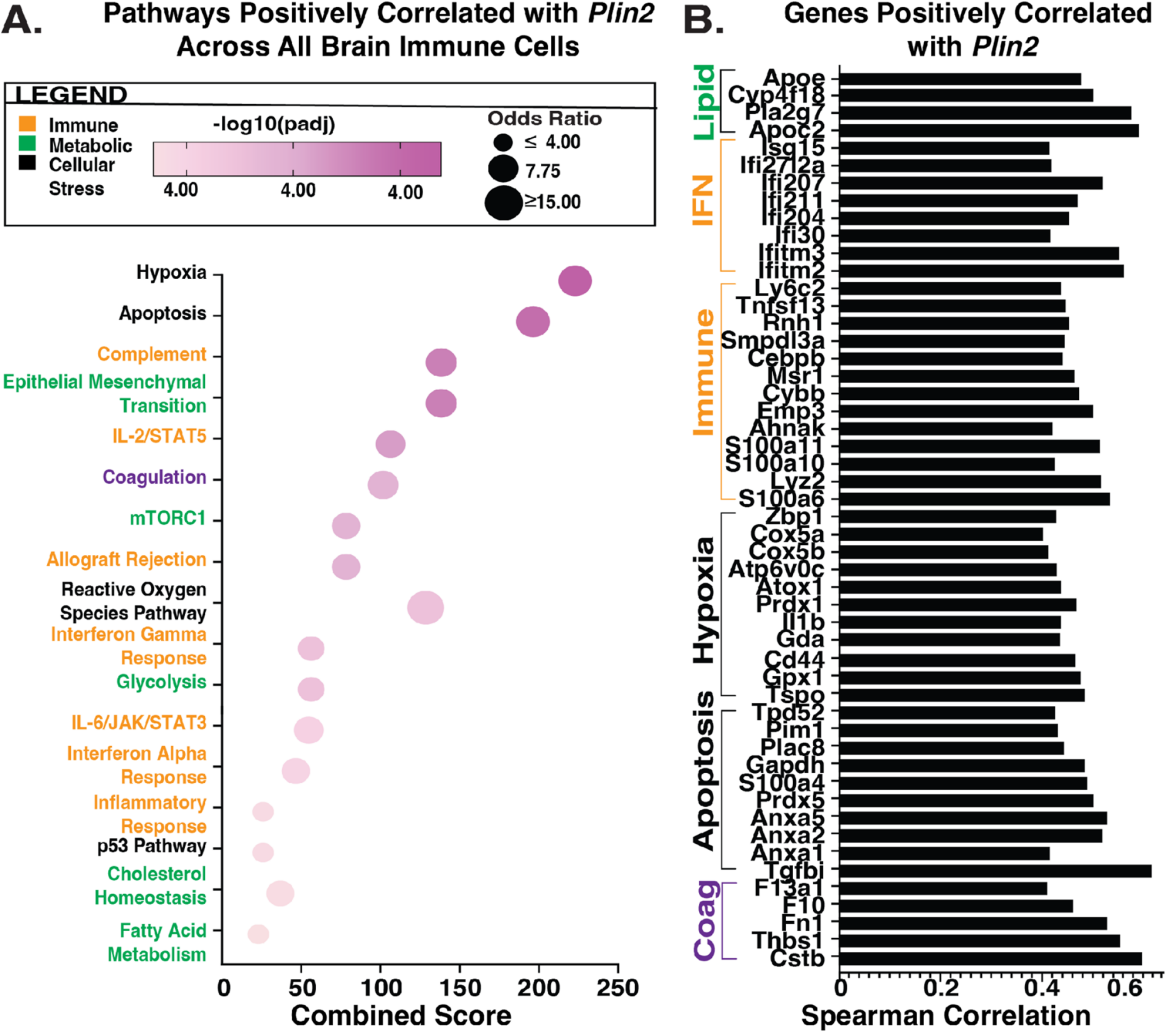
*Plin2* is positively correlated with genes involved in lipid biogenesis, inflammation, cellular stress, and coagulation. **A** Pathways positively correlated with *Plin2* expression across all brain immune cells. Genes included in the analysis have a Spearman correlation of 0.4 or higher. Coagulation is labeled in purple as a separate pathway. **B** Representative genes within pathways positively correlated with *Plin2* expression (IFN, interferon signaling; Coag, coagulation)

Given the consistent upregulation of lipid-associated genes across the blood and brain after stroke in obese mice, we next asked whether *Plin2*, a critical protein for lipid droplet accumulation and stability, might act as a central node linking lipid accumulation to broader immune, metabolic, and pro-thrombotic disturbances. To assess its connection to the other obesity-exacerbated processes we identified, we performed a Spearman correlation analysis centralizing *Plin2* expression across all brain immune cells. We found that genes involved in the lipid biogenesis, immune, oxidative stress, and coagulation pathways elevated in brain macrophages and neutrophils were positively correlated with *Plin2* (Fig. 5a). *Plin2* expression was at least moderately (Spearman Coefficient ≥ 0.4) and positively correlated with other genes involved in lipid biogenesis, interferon signaling, hypoxia, apoptosis, and coagulation (Fig. 5b). Notably, many of the genes that we observed to be elevated by obesity in brain macrophages and/or neutrophils such as *Pla2g7*,* Apoe*,* Ifitm2*,* Ifitm3*,* Isg15*,* Tspo*, and *Ctsb* are also correlated with *Plin2* across all brain immune cells (Fig. 5b) suggesting that it is co-regulated with major immune response changes in these cells.

### *Plin2* siRNA treatment worsens motor function after stroke compared to control siRNA in non-obese mice after stroke

Given that lipid-handling genes—particularly *Plin2*—were among the most highly upregulated genes in macrophages and neutrophils of obese mice after stroke, and that *Plin2* expression positively correlated with genes involved in not only lipid biogenesis, but also interferon signaling, oxidative stress, and cell death mechanisms, this raised two questions. First, is *Plin2* upregulation beneficial or harmful? Secondly, is *Plin2* driving the innate immune response that is supersized by obesity? To address these questions we utilized an intracortical injection of *Plin2* siRNA into the stroke core one day after stroke, when macrophages and neutrophils are infiltrating the stroke. We assessed motor behavior and stroke size, and neuroinflammation and oxidative stress using immunostaining for the lysosomal protein CD68 and the interferon-responsive IFITM3. We also chose to stain for TSPO, a cellular stress marker that is connected to immune activation within TNF and interferon-gamma signaling pathways and oxidative stress related to mitochondrial dysfunction [44–47].

**Fig. 6:**
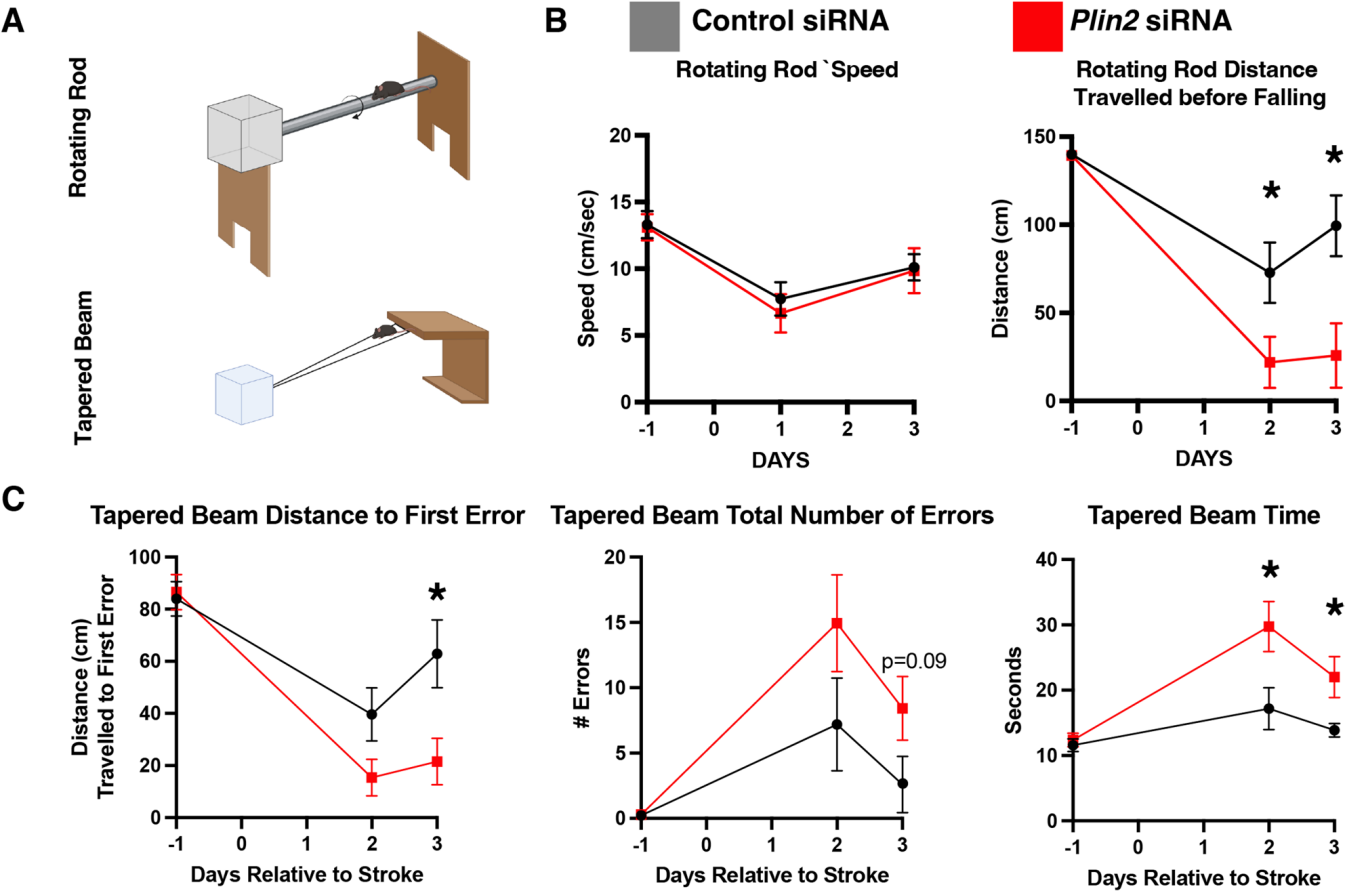
*Plin2* siRNA treatment worsened motor function after stroke (*n* = 8–9 mice). **A** Schematic of rotating rod (top) and tapered beam task (bottom) (**B**) Rotating rod task performance defined by distance mice traveled before falling (left) and mouse speed (right). **C** Tapered beam task performance defined by distance mice travelled when their left front paw slipped off the tapered beam for the first time (left), total number of left front paw slips (middle), and time it took for the mice to travel down the tapered beam (right). Error, SEM; Statistics, mixed linear model with Tukey’s post-hoc test; *p < 0.05

**Fig. 7:**
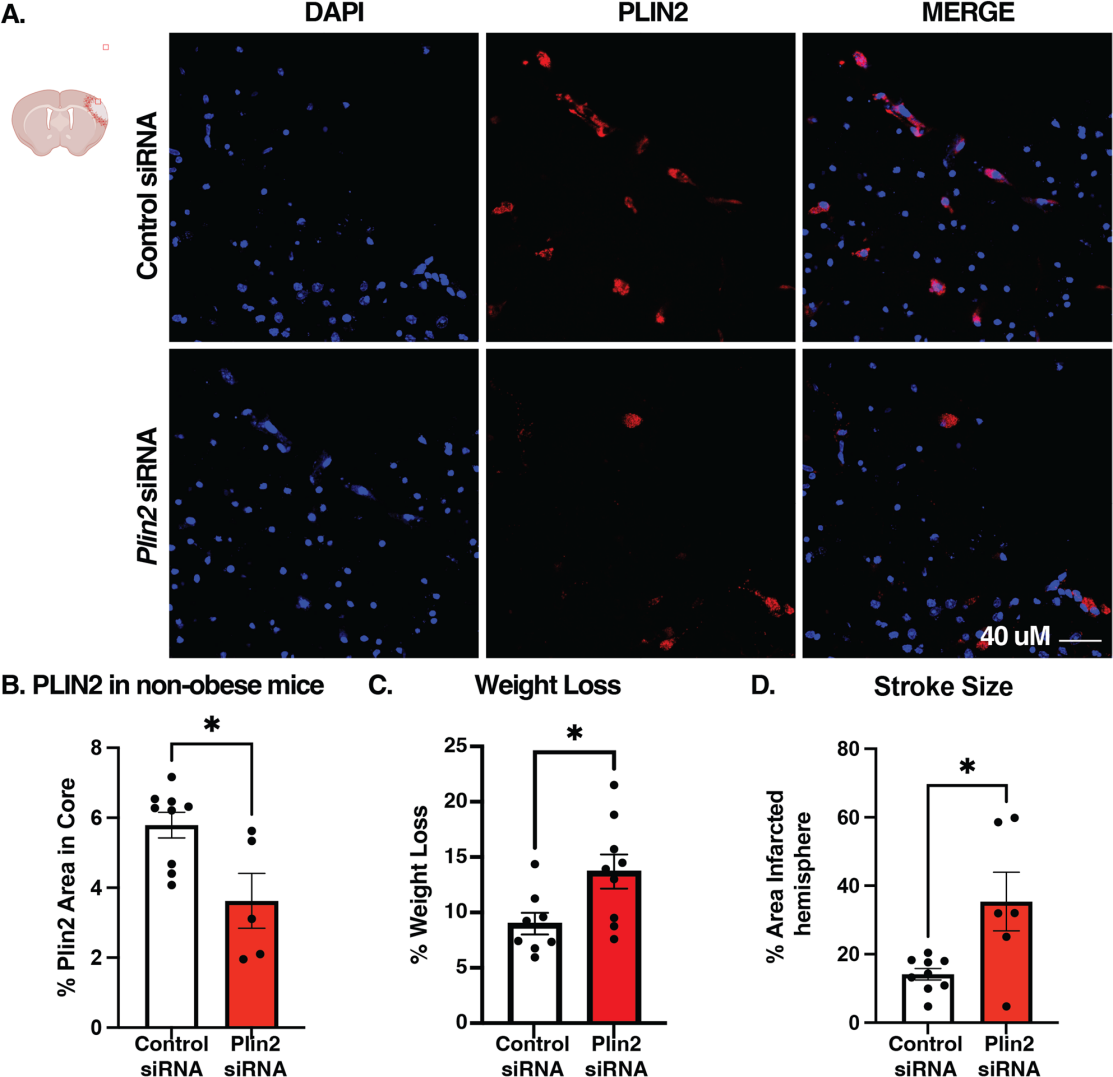
*Plin2* siRNA in non-obese mice knocks down PLIN2 and induces greater weight loss and stroke. **A**-**B** PLIN2 representative images and quantification at 3 days after stroke in non-obese mice with a 1 ug/uL siRNA treatment at 1 day after stroke (*n* = 5–9). **C** Weight loss of mice after stroke on day 3. **D** Quantification of stroke size established by lack of NEUN stain (*n* = 7 sections, *n* = 6–9 mice). Statistics, Student’s t-test; *p < 0.05; Bars, mean ± SEM

To evaluate the effects of *Plin2* siRNA treatment on motor outcomes in non-obese mice, we assessed performance using two behavioral tasks: the rotarod and the tapered beam (Fig. 6a). On the rotating rod, *Plin2* siRNA did not induce any differences in speed, but the distance at which the mice fell off the rod was shorter at days 2 (Fig. 7b; Control = 72.81 ± 51.44 cm, *Plin2* siRNA = 21.92 ± 41.08 cm; *p* = 0.0389) and 3 (Control = 99.46 ± 48.70 cm, Plin2 siRNA = 25.83 ± 51.69 cm; *p* = 0.0110) compared to control siRNA treatment (Fig. 6b).

On the tapered beam task, (Fig. 6c), the distance that the Plin2 siRNA-treated mice traveled before making the first slip was significantly reduced on Day 3 compared to mice injected with control siRNA (mean ± SEM: Control = 62.83 ± 36.81 cm, *Plin2* siRNA = 21.54 ± 25.335 cm; *p* = 0.0221). This was also reflected in the total number of errors which trended higher in *Plin2* siRNA-treated mice at day 3 (control siRNA mean = 2.58 ± 6.12 errors, *Plin2* siRNA mean = 8.42 ± 6.88 errors, *p* = 0.1). Finally, the *Plin2* siRNA-treated mice traveled significantly more slowly down the tapered beam compared to the control siRNA mice on days 2 (control siRNA mean = 17.17 ± 9.64 s, *Plin2* siRNA mean = 29.75 ± 10.86 s, *p* = 0.0247) and 3 (control siRNA mean = 13.88 ± 2.97 s, *Plin2* siRNA mean = 22.00 ± 8.87 s, *p* = 0.0377).

### *Plin2* siRNA knocks down PLIN2 and expands stroke size in non-obese mice

In non-obese mice, intracortical administration of *Plin2* siRNA (1 µg/µL) 24 h after dMCAO resulted in suppression of PLIN2 protein expression by 3 days after stroke by ~ 45% (*p* < 0.05, Fig. 7a; b). To determine the lethality of *Plin2* knockdown with siRNA, we compared survival after siRNA treatment. Though *Plin2* siRNA–treated mice did not die more after stroke (1 death out of 10 in control, 2 in 10 deaths in *Plin2* siRNA, *p* = 0.346), they did lose more weight and exhibited greater stroke sizes (Fig. 7c-e).

### *Plin2* siRNA reduces CD68, IFITM3, TSPO in non-obese mice

**Fig. 8:**
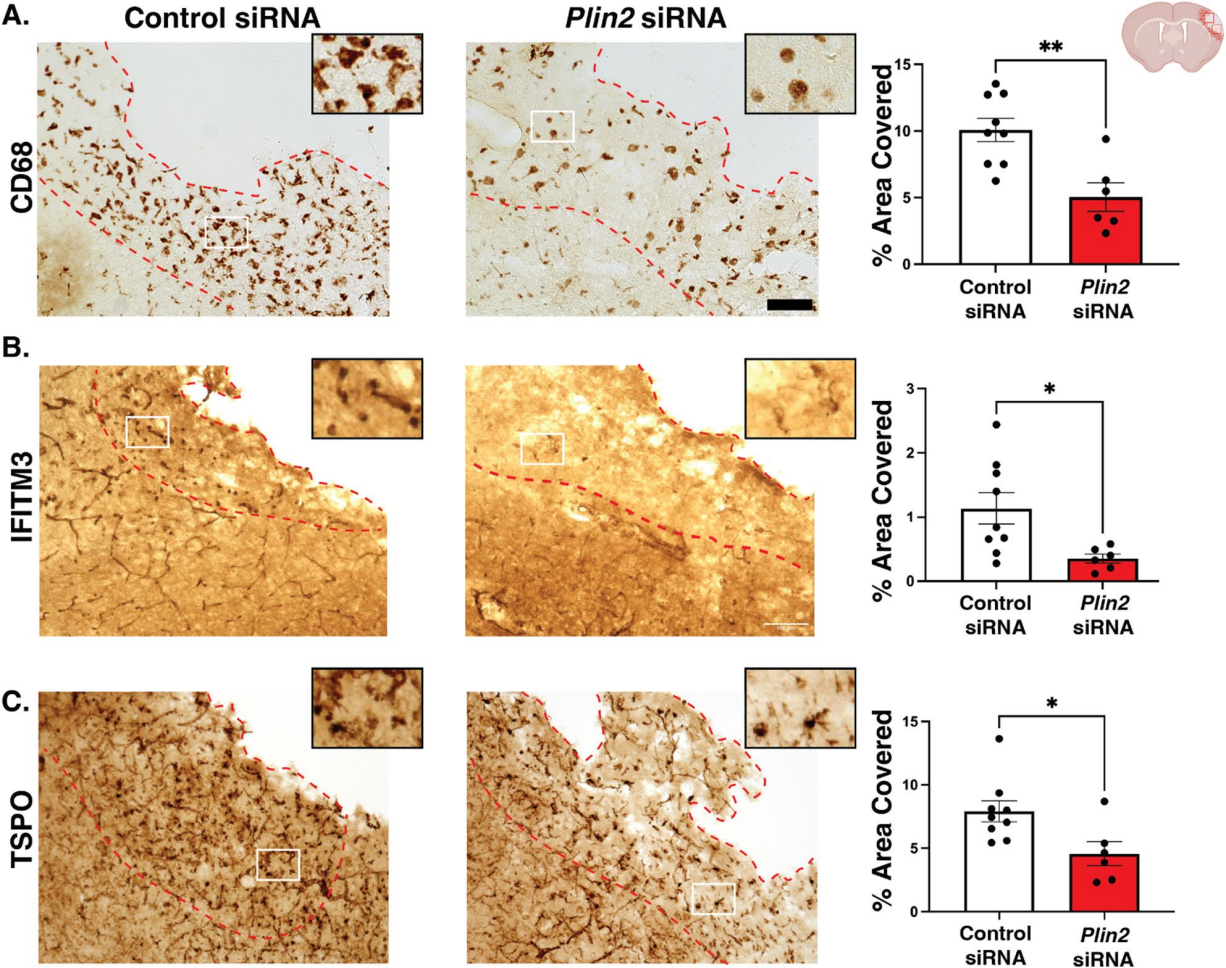
*Plin2* siRNA reduces CD68, IFITM3, and TSPO in the stroke core of non-obese mice. Normal diet-fed mice received 1 ug/uL siRNA that was scrambled or against *Plin2* 1 day after stroke and were sacrificed on day 3. **A** CD68 representative images (left) and quantification (right). **B** IFITM3 representative images (left) and quantification (right). **C** TSPO representative images (left) and quantification (right). The stroke core is outlined in red, and the location for the zoomed-in image (x4) is outlined in white. Scale bar, 100 μm; *n* = 5–10 animals, 2–3 sections per animal; Statistics, Student’s t-test; *p < 0.05; Bars, mean ± SEM

While stroke sizes and motor performance were worsened in non-obese mice treated with *Plin2* siRNA, the macrophage activation marker CD68, interferon signaling protein IFITM3, and and inflammatory and cellular stress marker TSPO all exhibited less expression, as measured by % area covered in the stroke core. The extent of the decrease was ~ 50%, similar to PLIN2 knockdown (Fig. 8, A-C).

### Plin2 siRNA worsens performance on the tapered beam task in obese mice

**Fig. 9:**
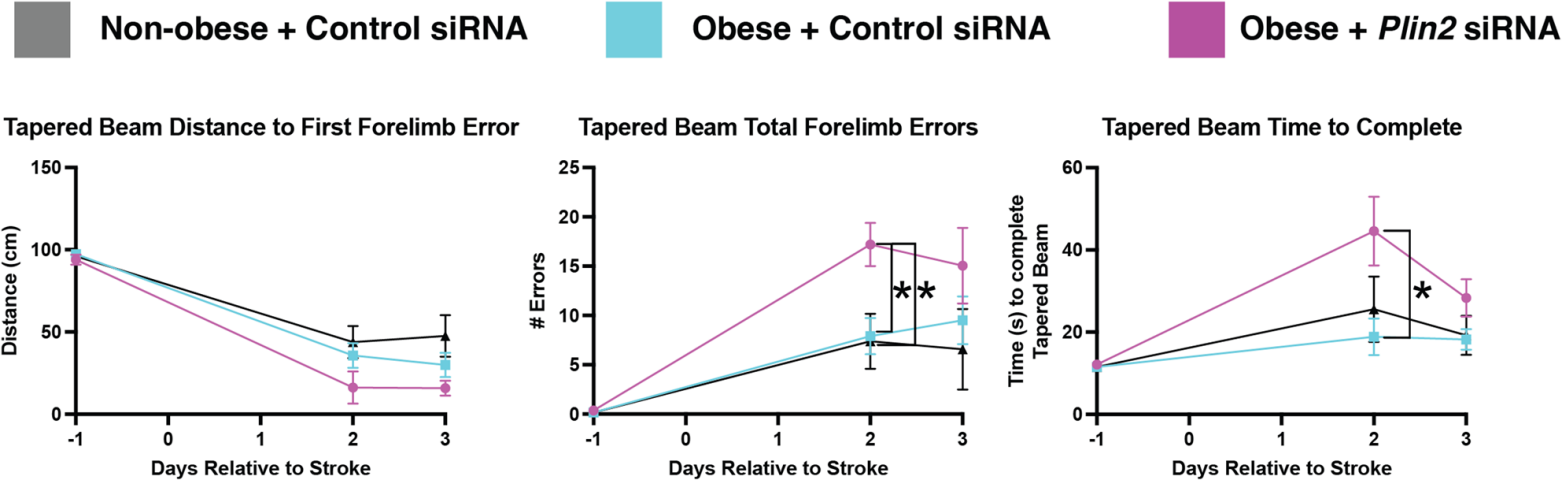
*Plin2* siRNA worsens performance on the tapered beam task in obese mice. Tapered beam task performance defined by distance mice travelled when their left front paw slipped off the tapered beam for the first time (left), total number of left front paw slips (middle), and time it took for the mice to travel down the tapered beam (right). Error, SEM; Statistics, mixed linear model with Tukey’s post-hoc test

After finding that knocking out *Plin2* in non-obese mice worsens functional outcomes, we next addressed the role of *Plin2* when it is elevated by obesity. In obese mice, the effect of *Plin2* knockdown on behavior was not as pronounced as in non-obese mice; it only significantly worsened performance on the tapered beam task on day 2 (*p* < 0.05) and did not alter performance on the rotating rod (Fig. 9, Additional File 10- Supplementary Fig. 8a). This is in spite of *Plin2* siRNA knocking down PLIN2 by ~ 45%, similar to the degree of downregulation in non-obese mice (Additional file 10-Supplementary Fig. 8b). Furthermore, death and weight loss were not affected by *Plin2* siRNA treatment in the obese mice, nor did stroke size change (Additional file 10-Supplementary Fig. 8c-e).

### Post-stroke *Plin2 *siRNA reduces CD68 in obese mice with no significant effect in IFITM3 or TSPO

**Fig. 10:**
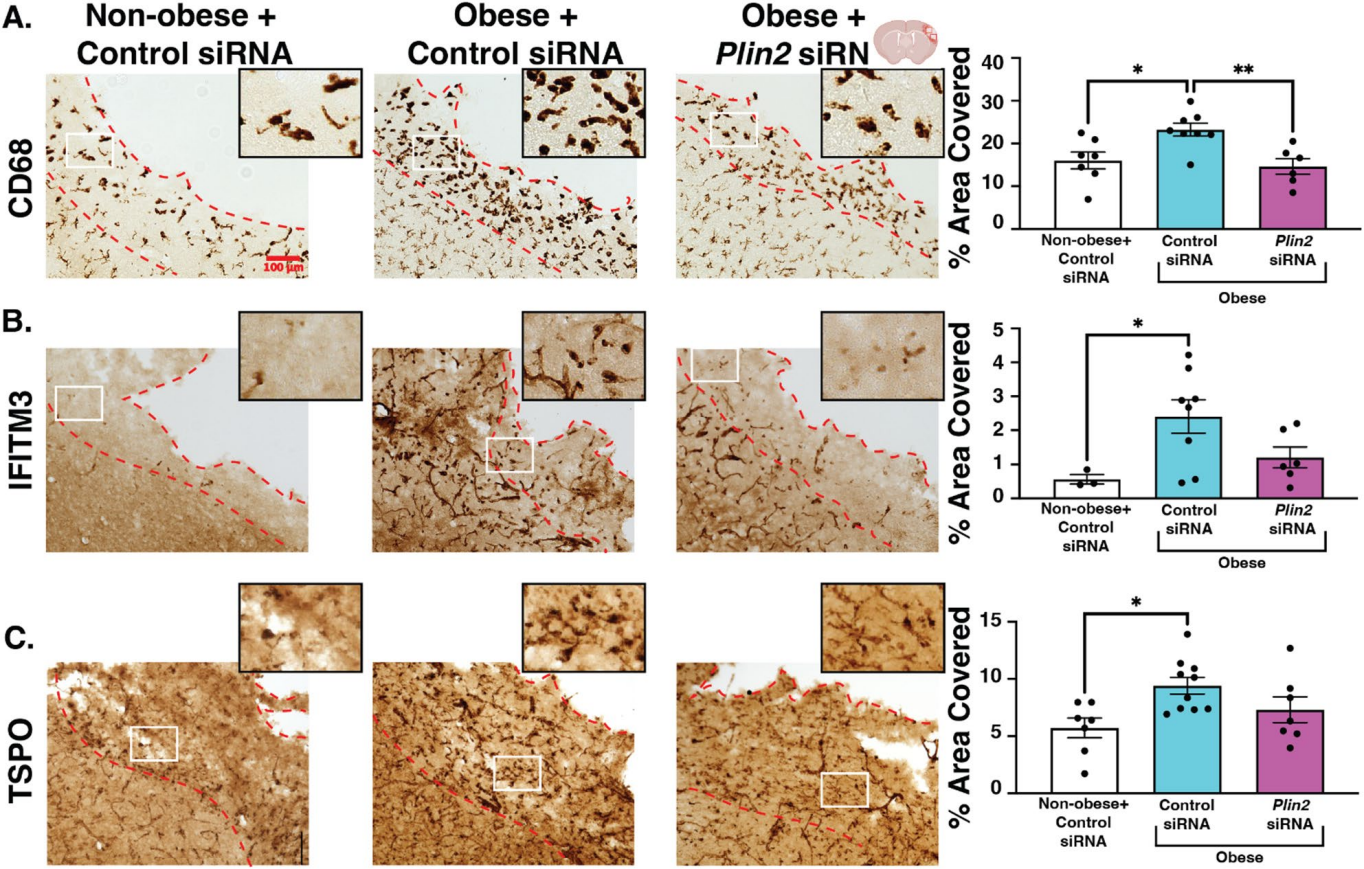
*Plin2* siRNA reduces CD68 to non-obese stroke level, but does not affect IFITM3 or TSPO immunostaining. All mice had stroke and received 1 ug/uL siRNA treatment at 1 day after stroke, either control or *Plin2* siRNA, then were sacrificed on day 3. **A** CD68 representative images (left) and quantification (right). **B** IFITM3 representative images (left) and quantification (right). **C** TSPO representative images (left) and quantification (right). The stroke core is outlined in red. Location for the zoomed-in image (x4) is outlined in white. *n* = 5–10 animals, 2–3 sections per animal; Statistics, Kruskal-Wallis with Tukey’s post-hoc test; *p < 0.05; **p < 0.001; Bars, Mean ± SEM

Compared to non-obese mice with stroke, as expected, we observed increased immunostaining for CD68, IFITM3, and TSPO in obese mice. This is commensurate with the gene upregulation we reported in the scRNA-seq study. In terms of % area covered in the stroke core, we saw elevated levels of CD68 (1.45-fold increase, *p* < 0.05), IFITM3 (6.38-fold increase, *p* < 0.05), and TSPO (1.65-fold increase, *p* < 0.05) compared to non-obese controls. As with non-obese mice, *Plin2* knockdown significantly reduced obesity-elevated CD68 expression in the stroke core of obese mice by 0.63-fold (*p* < 0.05). However, it did not significantly alter IFITM3 or TSPO (Fig. 10a–c).

## Discussion

In this study, we used high sequencing depth scRNA-Seq (average > 5,000 reads per cell) to examine the transcriptional effects of obesity on circulating and brain immune cells in a mouse model of diet-induced obesity and stroke. Our data reveal minimal obesity-induced transcriptional changes in immune cells from the brains of mice after sham surgery, but substantial gene expression changes after stroke, predominantly in macrophage and neutrophil subtypes. In Br.Macro.2, these changes point to the upregulation of lipid droplet generation, sustained interferon signaling, and elevated cellular stress marked by the loss of protein stability. In Br.Neut.1, inflammation defined by complement, TNF, and interferon alpha signaling was elevated by obesity along with signs of poor adaptation to cellular stress marked by genes consistent with dampened energy-production, reduced antioxidant capacity, and increases in apoptosis and hypoxia response. Genes in the coagulation pathway that are involved in platelet aggregation and activation were also upregulated in neutrophils. Among the most prominently upregulated genes was the lipid-binding gene *Plin2*, whose expression correlated with lipid biogenesis, inflammation, and cellular stress pathways. To test its role in early stroke outcomes, we knocked down *Plin2* using siRNA and found that this treatment worsened motor deficits in both non-obese and obese mice and increased stroke size in non-obese mice, supporting a protective role for lipid droplets early after stroke. Reduced CD68, IFITM3, and TSPO following *Plin2* knockdown further suggests that PLIN2 modulates immune activation, interferon signaling, and cellular stress responses in the injured brain. Our findings highlight mechanisms of obesity-exacerbated stroke outcomes that are not only congruent with previously reported changes seen in humans with obesity but also further characterize the roles of specific macrophage and neutrophil populations and lipid droplets in mediating the early immune response to stroke.

In the blood of mice after sham surgery, the obesity-induced changes we observed here are consistent with prior reports of enhanced lipid droplet accumulation and oxidative stress in obesity [11]. Elevated interferon signaling in macrophages, TNF signaling across all cell types, and complement signaling in neutrophils are corroborated by inflammatory changes seen in adipose tissue of rodent obesity models [13, 19, 20].

In contrast to the blood, the somatosensory cortex (where the stroke occurs in our model) of obese mice in the sham group demonstrated only a few differentially expressed genes compared to non-obese mice, primarily in microglial subtypes. Although limited in number, these differentially expressed genes are mainly heat shock and interferon signaling genes that align with prior work showing a comparable number of differentially expressed genes in the microglia of a similar region using single-nuclei RNA sequencing [48]. Our approach was designed to prioritize understanding of the local obesity–stroke interaction within the ischemic microenvironment. The cortical response to obesity may differ from the hippocampus, a brain region vulnerable in dementia that is particularly susceptible to metabolic diseases due to its high density of insulin receptors, enhanced blood-brain barrier permeability, and ongoing neurogenesis [49–52]. In this structure, obesity (without stroke) has been reported to increase hippocampal neuroinflammation [49, 53], blood–brain barrier permeability [54], dysfunctional neurogenesis [55], and memory impairment [49–51, 56, 57]. Thus, there could be additional important changes in the hippocampus of our mice that we did not observe as our single-cell analysis was intentionally focused on the cortical region immediately surrounding the infarct.

However, after stroke, obesity dramatically amplified immune activation in the brain. First, we saw that the proportion of the *Plin2*-high Br.Macro.2 macrophage subtype was approximately five times higher after stroke, regardless of obesity status. This is consistent with reports of elevated PLIN2 protein after transient ischemic stroke [58]. We added more depth to this analysis with evidence that in obesity, *Plin2* gene expression was doubled within these cells and reflected in protein levels seen on immunostaining. Confirming prior reports of obesity-elevated cytokine and chemokine signaling after stroke, we found that lipid-enriched Br.Macro.2 in obese mice mirrors changes we and others see in the blood after stroke with elevated interferon signaling [4, 25, 29]. Notably, the downregulation of interferon signaling in the macrophages of non-obese mice at 3 days after stroke supports prior reports that upon entering the brain after stroke, macrophages in non-obese mice adopt an initial anti-inflammatory profile that gradually becomes more pro-inflammatory by 14 days [59–61], later than we studied here. This initial response is dampened by obesity, perhaps causing sustained inflammation and protein instability given elevated proteasome and reduced protein translation genes within the Myc V1 targets pathway.

Given the antioxidant role of lipid droplets in sequestering active lipids and mitigating oxidative stress in the physiological state [62–64], perhaps *Plin2*-high macrophage subtypes normally suppress interferon signaling in response to stroke but lose this capacity with obesity in a process that occurs separately from lipid metabolic changes or in response to an excess in lipid droplets or lipids, as that has also been shown to drive oxidative stress [65–67]. We did indeed observe an association between interferon pathway increases and lipid droplets, as the changes we describe are not seen in the Br.Macro.3 subtype that has lower *Plin2* expression. And our knockdown of *Plin2* resulted in less expression of the interferon protein IFITM3, implying that *Plin2* is upstream of interferon signaling.

While obesity exacerbated inflammation and oxidative stress in blood and brain macrophages after stroke, we found that obesity polarized responses to stroke in brain neutrophils. Specifically, in non-obese mice with stroke, complement signaling trends towards a decrease in response to stroke, but increases after stroke in obese mice. In contrast, oxidative phosphorylation pathway genes related to ATP synthesis, glycolysis, and antioxidant genes were significantly upregulated in non-obese mice after stroke but downregulated or unchanged in obese mice. Given that neutrophils in obese mice lose the ability to respond to the metabolic demands of cancer as key energy processing and antioxidant genes are suppressed [68], there may be a similar issue occurring after stroke. We suspect that these changes exacerbate neuroinflammation and compromise metabolic resilience after stroke, driving increased cell death.

Our data also confirms that neutrophils likely contribute to thromboinflammation in obese mice. Others have found that neutrophils in obese mice do expand stroke size via NET release mediated by elevated fibrinogen [69]. Our data suggests that this process may be exacerbated in obese mice as elevated complement signaling and coagulation-related genes drive NET formation [70]. In the blood, neutrophils in obese mice display enhanced *F5* along with the NETosis marker *Padi4*, and in the brain coagulation-related genes *Ctsb*,* Ctsd*,* Calr and Cd63* are elevated. *Ctsb* and *Ctsd* are extracellular matrix degradation genes that drive coagulation by exposing tissue factor [71]. Notably, *Calr* is a diagnostic biomarker for essential thrombocythemia and considered to be a risk factor for stroke due to its role in platelet production [72–74]. The elevation of coagulation genes we define suggests that in addition to NETosis, platelet aggregation driven by neutrophils is also contributing to blood clots and this could contribute to stroke size expansion.

Lipid droplet accumulation is known to drive inflammation, oxidative stress, and coagulation in obesity and hyperlipidemia [75–77], all generally thought to be harmful. Indeed, our co-expression analysis showed that *Plin2* was positively correlated not only with other lipid droplet-associated genes but also with genes involved in inflammation, cellular stress, and coagulation. Specifically, the elevation of interferon signaling in Br.Macro.2 could align with lipid droplets acting as hubs for interferon-γ signaling [15, 16, 78, 79], activating precursor toll-like receptor signaling [80], and reciprocally promoting further lipid droplet formation [81–84]. In the context of stroke, these interactions could promote a more pro-inflammatory and stressed phenotype in the lipid-laden macrophages of obese mice. However, there was also the possibility that lipid droplets regulate inflammatory and cellular stress pathways in a protective role. In the physiological state, lipid droplets function to protect cell membranes from lipid peroxidation by sequestering toxic lipids and regulating oxidative stress [16, 62, 63]. This could be crucial early after stroke when free fatty acids and cholesterol esters from myelin and cellular debris are released.

Indeed, our knockdown experiments demonstrate that *Plin2* upregulation is beneficial in the days after stroke, particularly in non-obese mice. We found that motor behavior was worse and stroke sizes were larger, suggesting that PLIN2 is critical for neuroprotection early after stroke. Additionally, CD68 and markers of interferon signaling (IFITM3) and oxidative stress (TSPO) were reduced following *Plin2* knockdown. As PLIN2 protein is required to form lipid droplets [76, 77, 85], this suggests that early lipid droplet loss may impair cellular lipid buffering capacity and beneficial immune reactivity. This could then increase vulnerability to lipotoxicity and metabolic stress during the acute injury phase. The concept of beneficial immune reactivity is supported by prior studies that found that the non-selective depletion or depletion of anti-inflammatory macrophages worsen outcomes [86–88]. Or conversely, it could be that early depletion of these *Plin2*, lipid-handling cells worsens outcomes simply because free lipids are no longer sequestered. Indeed, our data is consistent with others who found that inhibiting lipid droplet breakdown starting 1 day after transient ischemic stroke reduces stroke size and enhances motor function on day 3, implying that lipid droplets are beneficial when augmented [89].

Although the direction of change following *Plin2* siRNA treatment in obese mice generally paralleled that observed in non-obese mice, the effects were not as dramatic. In obese mice, *Plin2* knockdown significantly worsened tapered beam performance and reduced obesity-elevated CD68 levels, despite achieving a comparable percentage reduction in PLIN2 signal within the infarct core. Regardless, these findings are clearly not consistent with a harmful role of PLIN2 in subacute stroke in obese mice.

This dampened functional response in the obese mice to *Plin2* siRNA may be due to an insufficient knockdown of *Plin2* in the obese mice or we may have been underpowered to detect a behavioral worsening. More broadly, these findings raise the possibility that certain obesity-associated adaptations may confer context-specific benefits following acute injury. It is possible that there are other conditions under which obesity-driven changes are beneficial. The so-called “obesity paradox” is that some but not all studies demonstrate better outcomes in obese populations [90–92]. Our results with *Plin2* knockdown are consistent with this. It may be that increased *Plin2* is one benefit to obese stroke patients, who may have more capacity to accommodate lipid droplets within macrophages, and that this may mitigate the additional lipotoxicity from the stroke-induced increase in myelin lipids. It is currently challenging to decipher true outcomes of human stroke as clinical studies are nuanced and may be influenced by factors like stroke type, age, obesity diagnosis, and experimental timeframe [90, 91]. However, given the congruence of our model with human data we hope that future studies will shed light on whether there are beneficial components in the obesity-stimulated immune response to stroke.

Together, our scRNA-seq data and PLIN2 intervention are consistent with a protective early role of PLIN2 but our study did not address later effects of lipid droplets, which could be detrimental. Persistent lipid droplet accumulation—especially in obesity—may transition from adaptive buffering to maladaptive signaling. Chronic lipid enrichment in monocytic cells has been linked to sustained interferon and TNF signaling [82–84, 93, 94], impaired inflammatory resolution [93, 95], and lipotoxicity-driven macrophage polarization and inflammasome activation. Chronic neuroinflammation downstream of lipid accumulation is associated with increased blood–brain barrier permeability [96], immune cell infiltration [28, 33, 97], neuronal cell death [28, 33, 97], and larger stroke sizes in obese mice. Obesity-primed immune cells can degrade tight junction proteins and activate matrix metalloproteinases, compromising vascular integrity as observed in obesity and multiple sclerosis [89, 98–101]. A weakened barrier may then permit further infiltration of lipid-laden peripheral immune cells [102, 103], reinforcing a feed-forward inflammatory loop. In parallel, oxidative stress linked to lipid droplet accumulation—described in aging microglia with disease-associated transcriptional signatures [104]—may exacerbate mitochondrial dysfunction, deplete antioxidant reserves, and promote apoptosis [105, 106]. These reported changes in processes mirror the pathways amplified in our study, including interferon responses, oxidative stress, and pro-coagulant programs. Lipid-laden “foamy” macrophages, described in other inflammatory diseases, similarly exhibit both protective lipid sequestration and pathogenic inflammatory potential, underscoring the duality of this biology. It may be that a stage-specific strategy will be useful, with acute preservation of lipid droplet integrity to maintain metabolic buffering, followed by later-phase modulation of chronic lipid accumulation that sustains inflammatory and thromboinflammatory signaling. Becktel et al. 2024 demonstrated that in non-obese mice, blocking lipid-droplets associated with myelin degradation and cellular debris 1 week after stroke mitigates chronic neuroinflammation and improves outcomes [107].

Encouragingly, the pathways we define related to neuroinflammation, oxidative stress, and coagulation that we have identified are also strong therapeutic targets for stroke in obese populations. Similarly to lipid droplet interventions, blocking interferon or complement signaling after stroke prevents cell death in oxygen glucose deprivation, inflammation, and stroke size in non-obese mice [108]. Oxidative stress is also a viable therapeutic candidate with studies demonstrating that suppressing reactive oxygen species production or neutralizing it with antioxidants can reduce stroke size and improve functional outcomes [109, 110]. Complementary assays—including puromycin-based translation assays, ribosome profiling, and proteasome activity measurements—should be used in the future to determine whether observed transcriptional signatures translate into altered protein synthesis and proteostasis dynamics. Finally, targeting the enhanced coagulation processes that we see in brain neutrophils reduces stroke sizes [111–113]. However, any therapeutic approach will be critical to optimize for obese individuals as anticoagulants increase the risk of bleeding and obese patients exhibit differences in absorption and metabolism of anticoagulants [114, 115]. Altogether, the demonstrated benefits of targeting lipid droplets, inflammation, oxidative stress, and coagulation after stroke in non-obese mice warrants future studies that clarify which of these mechanisms hold the most therapeutic promise to target for obese stroke patients.

This study has several limitations. First, our scRNA-seq analysis was restricted to CD45 + immune cells, and therefore did not capture obesity-induced alterations in other critical cell populations such as astrocytes, endothelial cells, and neurons. Future studies should examine how immune–glial–vascular interactions collectively shape stroke pathology in metabolic disease. We also did not include naive controls, but our prior bulk RNA-sequencing study using the same diet-induced obesity and dMCAO model [21] demonstrated minimal transcriptional differences between naïve obese and non-obese mice, as we saw here with sham-operated mouse controls. There, we also reported similar elevation of interferon signaling and inflammatory pathways in obese mice three days after stroke. These findings support our use of sham-operated mice as appropriate controls for defining obesity-specific gene expression changes within the stroke context. Additionally, the mice we used (11–18 weeks) are relatively young and future studies should explore obesity and aging together. While these mice may not represent the majority of stroke survivors who are elderly, their obesity status could serve as a representation of 18 to 44% of stroke patients who are obese as well as the 63% of young people with stroke who are also obese [2, 116].

In addition, samples were pooled from multiple animals, limiting statistical power in quantitatively comparing cell proportion changes and our ability to assess inter-animal variability in this study. To address this shortcoming, we performed immunohistochemical analyses of PLIN2 and inflammatory markers (CD68, IFITM3, and TSPO) in multiple animals age-matched to those used in the scRNA-seq study. The changes we found at the protein level in these studies validate the key obesity-induced transcriptional changes identified by single-cell RNA sequencing. Additionally, our prior work using bulk RNA sequencing in the same diet-induced obesity and stroke model [21] demonstrated increased expression of several immune and lipid-associated genes, including *Plin2*,* Ifitm3*,* Ifit1*,* Irf7*,* Irf8*,* Isg15*,* C1qa*,* C1qb*,* C1qc*,* Tnf*,* Ccl2*,* Nos1*, and *NfκB* in individual animals (*n* = 3–6 animals). We also validated increased expression of *Tnf*,* Ccl2*,* Nos1*, and *NfκB* by qPCR (*n* = 6–10 animals) in that paper. We also previously demonstrated that obesity increases circulating levels of TNF, IL-6, IL-1β, IL-10, and IL-2 at 7 days after stroke [24]. These findings are consistent with our current scRNA-seq data showing enhanced TNF and interleukin signaling in macrophages and neutrophils in both blood and brain at 3 days post-stroke. Ultimately, the single-cell analyses we offer in this work extend our prior findings of elevated inflammation by resolving the cellular sources of these obesity-associated responses, highlighting macrophages and neutrophils as key contributors in both blood and brain after stroke.

The translational relevance of our findings in the brain is supported by human studies. Although single-cell datasets directly profiling human stroke tissue in the context of obesity remain limited, emerging human scRNA-seq studies in obesity (including adipose tissue and peripheral blood) consistently identify heightened interferon-driven immune states that parallel those observed here [117]. Consistent with our findings in our diet-induced obesity mouse model, interferon α/γ, TNF signaling, and both classical and alternative complement pathways are elevated immune pathways in obese humans across blood, liver and adipose tissue [18, 48, 118]. In macrophages, neutrophils, and dendritic cells in obese human adipose tissue, BMI is positively correlated with pro-inflammatory gene expression [4]. Obese individuals exhibit elevated interferon, TNF, and complement signaling in lipid-enriched immune cell populations [18, 118]. Evidence of oxidative stress in higher advanced oxidation protein products and lower antioxidant capacity are also observed in obese people [119]. Finally, coagulation activity in plasma is positively correlated with BMI and is reversed with weight loss [120–122].

By integrating high-depth single-cell profiling of blood and brain immune compartments with mechanistically rigorous *Plin2* downregulation, this work establishes a framework to define how obesity reprograms immune metabolism after stroke. Such an approach moves beyond descriptive inflammation toward identifying metabolically actionable pathways that shape injury progression and recovery. Continued investigation will refine our understanding of lipid droplet biology in neuroinflammation and enable the rational design of metabolically informed interventions. Our data suggest that indiscriminate early suppression of lipid droplet formation may be detrimental. Instead, therapeutic strategies that bolster lipid droplet integrity during the acute injury phase may be protective. Selective targeting of downstream immune pathways (including interferon signaling, complement activation, oxidative stress, and coagulation cascades) may also provide greater clinical benefit, and our online Shiny app (https://buckwalterlab.shinyapps.io/scrnaseq_hfd_shiny_app/) is designed to aid in exploring expression of additional candidate targets. Overall, this work will help to develop precision therapies aimed at improving stroke outcomes in obese patients.

## Supplementary Information

Additional file 1. Supplementary Table 1. FACsorted and sequenced cell numbers from each condition. Description: 3 mice were pooled into one sample per condition to be FACsorted for a maximum of 60,000 cells.

Additional file 2. Supplementary Table 2. Blood and Brain Cell Subtype Labels. Description: Identification of immune cell subtype clusters in blood (A) and brain (B). Major immune cell types were manually assigned based on CellMarker 2.0 database and prior literature. Subtypes within each major lineage (e.g., neutrophils, macrophages, dendritic cells, microglia) were further characterized by gene expression profiles indicative of specialized functions based on prior work. The top 20 genes enriched in each subtype (relative to the full blood or brain dataset) are listed. References used for defining major and subtype identities are provided.

Additional file 3. Supplementary Figure 1. Predicted doublets do not alter biological interpretations. Description: Correlation of log₂FC values for all differentially expressed genes (FDR < 0.05) in Bl.Neut.1 (left) and Br.Macro.2 (right) comparing analyses performed with and without predicted doublets identified by DoubletFinder. Pearson’s correlation coefficient for both immune cell subtypes exceeds 0.9, indicating high agreement. In both approaches, Eno1b and Acot1 remain among the most significantly affected genes, confirming that doublet handling does not meaningfully change the biological conclusions.

Additional file 4. Supplementary Figure 2. Metabolic changes in response to a high fat diet. Description: (A-B) Curve and area under the curve for the glucose tolerance test. Welch’s T-test used to compare area under the curve (*p<0.05) (C-D) Weight over time and percent of weight gain between week 0 and week 6 of the high fat diet. Mann-Whitney test was used to compare percentage of weight gain (** p<0.001).

Additional file 5. Supplementary Figure 3. Obesity-induced transcriptional changes in blood macrophages and neutrophils in mice after sham or stroke surgery. Description: Volcano plots showing differentially expressed genes induced by obesity in blood macrophages and neutrophils from mice after sham (A) and stroke (B) surgeries. Log2fc cut-off=|0.5| and fdr=0.05. Lipid-related genes are labeled in green. (C) MAP of blood and brain top affected macrophages.

Additional file 6. Title: Supplementary Figure 4. Average *Plin2* gene expression after stroke. Description: Average gene expression of *Plin2* in top changing blood and brain immune cells. ****- p<0.00001in MAST differential gene expression analysis. NO, non-obese; Ob, obese.

Additional file 7. Supplementary Figure 5: Oil Red staining is elevated in obese mice compared to non-obese mice three days after stroke. Description: Quantification (left) and representative images (right) of oil red in stroke core (40X objective, n=3 sections/mouse, n=4-5 mice/group). Student t-test was used for statistical comparison (* p<0.05).

Additional file 8. Supplementary Figure 6. Top obesity-enriched pathways in blood macrophages in mice after sham surgery. Description: Top obesity-changed pathways in Bl.Macro.1 (left) and Bl.Macro.2 (right) of mice after sham surgery. Pathways are from the MSigDB Hallmark 2020 database (fdr < 0.05).

Additional file 9. Supplementary Figure 7. Top obesity-enriched pathways and coagulation gene changes in blood neutrophils in mice after sham surgery. Description: Top obesity-changed pathways in Bl.Neut.1 (left), Bl.Neut.2 (middle), and Bl.Neut.3 (right) of mice after sham surgery. Pathways are from the MSigDB Hallmark 2020 database, genes used had fdr < 0.05. (B) Heatmap of classic coagulation genes changed by obesity after stroke in blood neutrophils.

Additional file 10. Supplementary Figure 8. Plin2 siRNA does not change weight loss or rotating rod performance in obese mice. Description: (A) Rotating Rod task performance defined by distance mice traveled before falling (left) and mouse speed (right). SEM bars are displayed. * p-value <0.05 in mixed model, Tukey’s post-hoc analysis (​​n=8 for non-obese mice, n=14-16 for obese mice). (B) Representative PLIN2 fluorescence image in the stroke core of obese mice (left) and quantification(right). (C) Death curve after stroke surgery (D) Weight loss of mice after stroke on day 3. (E) Quantification of stroke size established by lack of NEUN stain (n=7 sections, n=6-9 mice). Statistics, Student’s t-test; *p < 0.05; Bars, mean ± SEM.

## Data Availability

The dataset(s) supporting the conclusions of this article are available in NCBI [#GSE310324] and hyperlink to dataset(s) is at https://www.ncbi.nlm.nih.gov/geo/query/acc.cgi?acc=GSE310324.

## Abbreviations

scRNA-Seq: Single-cell RNA sequencing
Plin2: Perilipin-2
TNF: Tumor necrosis factor
dMCAO: Distal middle cerebral artery occlusion
dPBS: Dulbecco's phosphate-buffered saline
FACs: Fluorescence Activated Cell sorting
PBMCs: Peripheral Blood Mononuclear Cells
