# Supplementary material for: Obesity supersizes macrophage and neutrophil activation after stroke while lipid droplets play a protective role
Source: J Neuroinflammation. 2026 Mar 19;23:158. doi: 10.1186/s12974-026-03774-7 (PMC13188555; doi:10.1186/s12974-026-03774-7)
Supplement: Supplementary file 1 — Additional file 1. Supplementary Table 1. FACsorted and sequenced cell numbers from each condition. Description: 3 mice were pooled into one sample per condition to be FACsorted for a maximum of 60,000 cells. [file 12974_2026_3774_MOESM1_ESM.docx]

**Supplementary Table 1.** FACsorted and sequenced cell numbers from each condition. 3 mice were pooled into one sample per condition to be FACsorted for a maximum of 60,000 cells.

| **Condition** | **Tissue** | **# FACsorted Cells** | **#Cells placed on the 10x sequencer** | **#Analyzed Cells** |
| --- | --- | --- | --- | --- |
| **Non-obese Sham** | **Blood** | **60,000** | **7,558** | **7,486** |
| **Obese Sham** | **Blood** | **60,000** | **7,725** | **7,519** |
| **Non-obese Stroke** | **Blood** | **60,000** | **6,083** | **5,722** |
| **Obese Stroke** | **Blood** | **56,750** | **6,738** | **6,710** |
| **Non-obese Sham** | **Brain** | **12,896** | **5,650** | **5,477** |
| **Obese Sham** | **Brain** | **10,752** | **5,398** | **5,236** |
| **Non-obese Stroke** | **Brain** | **60,000** | **12,082** | **11,068** |
| **Obese Stroke** | **Brain** | **60,000** | **13,405** | **12,026** |
