# Supplementary material for: Obesity supersizes macrophage and neutrophil activation after stroke while lipid droplets play a protective role
Source: J Neuroinflammation. 2026 Mar 19;23:158. doi: 10.1186/s12974-026-03774-7 (PMC13188555; doi:10.1186/s12974-026-03774-7)
Supplement: Supplementary file 2 — Additional file 2. Supplementary Table 2. Blood and Brain Cell Subtype Labels. Description: Identification of immune cell subtype clusters in blood (A) and brain (B). Major immune cell types were manually assigned based on CellMarker 2.0 database and prior literature. Subtypes within each major lineage (e.g., neutrophils, macrophages, dendritic cells, microglia) were further characterized by gene expression profiles indicative of specialized functions based on prior work. The top 20 genes enriched in each subtype (relative to the full blood or brain dataset) are listed. References used for defining major and subtype identities are provided. [file 12974_2026_3774_MOESM2_ESM.docx]

**Additional file 2. Supplementary Table 2. Blood and Brain Cell Subtype Labels.** Identification of immune cell subtype clusters in blood (A) and brain (B). Major immune cell types were manually assigned based on CellMarker 2.0 database and prior literature. Subtypes within each major lineage (e.g., neutrophils, macrophages, dendritic cells, microglia) were further characterized by gene expression profiles indicative of specialized functions based on prior work. The top 20 genes enriched in each subtype (relative to the full blood or brain dataset) are listed. References used for defining major and subtype identities are provided.

**A. Blood**

| **Cell Type and Markers** | **Subtype** | **Potential Function (enriched genes that align with function)** | **Top 20 enriched genes in subtype** | **References** |
| --- | --- | --- | --- | --- |
| Neutrophils (Ly6g, S100a8, S100a9, Cxcr2) | Bl.Neut.1 | **Chemotaxis** (Cxcl2, Ccr1.7, Cxcr4.1, Fpr2, Mmp9, Ptfafr, Itgam, Itgab2, Rac2, Gm2a, Lst1, Fgl2, Itga4, Ptafr, Ppt1, Wfdc17) | Il1b, Csf3r, Neat1, Dusp1, Fgl2, Cxcr2, Tnfaip2, Marcks, Sorl1, Slc40a1, Gcnt2, Igf1r, H2-Q10, Ccr1, Nfam1, Slpi, Fos, Trim30b, Mxd1 | [(1)](https://paperpile.com/c/mmaqtZ/tVXb) |
|  | Bl.Neut.2 | **Chemotaxis 2** (Retnlg, Wfdc21, Wfdc17, Lcn2, Mmp8, Mmp9, Il1f9, Ccl6, Cxcr2, Ccr1) | Retnlg, Wfdc21, Lcn2, S100a9, S100a8, Mmp8, Ly6g, Wfdc17, Ifitm1, Cstdc4, S100a6, Lrg1, Mmp9, Pglyrp1, Chil1, S100a11, Asprv1, Grina, Cxcr2 | [(1)](https://paperpile.com/c/mmaqtZ/tVXb) |
|  | Bl.Neut.3 | **Interferon Signaling** (Ifit1, Isg15, Oasl2, Rsad2, Ifit3, Ifit2b, Ifit1, Irf7, Gm13822, Cmpk2) | Isg15, Rsad2, Ifit3, Ifit1, Slfn4, Slfn5, Oasl2, Ifit3b, Rtp4, Stfa2l1, Irf7, Oas3, Slpi, Oasl1, Ifi204, Il1b, Gbp2, Ddx60, Isg20, Ifitm1 | [(1)](https://paperpile.com/c/mmaqtZ/tVXb) |
|  | Bl.Neut.4 | **Anti-microbial** (Camp, Ltf, Lcn2) | Camp, Ngp, Ltf, Lcn2, Cd177, Ifitm6, Mmp8, Adpgk, Synei, Wfdc21, S100a8, S100a9, Ly6g, Anxa1, Ckap4, Dstn, Pglyrgp1, Retnlg, Mmp9, Itg2l | [(1–6)](https://paperpile.com/c/mmaqtZ/tVXb+kd8du+QOehi+57RIU+1Y7Cw+PIih) |
| Cd8+ T Cells (Cd3d, Cd3g, Cd3e, Cd8a, Cd8b1, Il7r, Trbc1, Trbc2) | Bl.Cd8+ T Cells.1 | | Cd8b1, Cd3d, Trbc2, Dapl1, Cd3g, Il7r, Rpl12, Rplp1, Rps18, Ms4a4b, Cd3e, Rpl36a, Rps15a, Rps20, Cd8a, Rpsa, Rpl32, Rps7, Trbc1, Rpl3 | [(6)](https://paperpile.com/c/mmaqtZ/PIih) |
|  | Bl.Cd8+ T Cells.2 | | Ms4a4b, Trbc1, Il7r, Cd3g, Ccl5, Trbc2, mt-Atp6, mt-Co3, mt-Cytb, H2-Q7, Bcl11b, mt-Nd2, Cd3e, Trac, Thy1, Lck, mt-Co2, mt-Nd1, Cd3d, Cd8b1 | [(6)](https://paperpile.com/c/mmaqtZ/PIih) |
|  | Bl.Cd8+ T Cells.3 | | Ms4a4b, Trbc1, Trbc2, Cd8b1, Il7r, Cd3d, Gm12840, Lef1, Cd3g, Tcf7, Thy1, Lck, Igfbp4, Bcl11b, Ccl5, Nkg7, Txk, Cd3e, Dapl1, Ets1 | [(6)](https://paperpile.com/c/mmaqtZ/PIih) |
|  | Bl.Cd8+ T Cells.4 | | Trbc2, Igfbp4, Lef1, Cd3d, Cd3g, Trbc1, Trac, Rpl12, Il7r, Dapl1, Tcf7, Cd3e, Gm12840, Ms4a4b, Ccr7, Rplp1, Bcl11b, Rpl5, Rps20, Rps7 | [(6)](https://paperpile.com/c/mmaqtZ/PIih) |
| Macrophages (Cd68, Adgre1, Ly6c2, Lyz2, Cx3cr1) | Bl.Macro.1 | **Monocyte-derived Non-foamy Plin2+** (Ly6c2, Ccr2, Cd68, Cx3cr1,  Lyz2, Adgre1, Plin2+/Cd36-) | Chil3, Lyz2, Plac8, S100a4, Ms4a6c, Ccr2, Fn1, Ifi30, F13a1, Lgals3, Ms4a4c, Ctss, Ms4a6d, Ctsc, Crip1, Dbi, Ly6c2, Ifitm3, Npc2, Ccl9 | [(7–14)](https://paperpile.com/c/mmaqtZ/UZbOW+Uqaw5+h5AZH+ALEoM+mOpo+r5WD+TDND+3yKn) |
|  | Bl.Macro.2 | **Foamy Plin2+ 1** (Cd68, Cx3cr1, Adgre1, Plin2, Cd36, Apoe, Apoc2, Fabp4, Ear2) | Gngt2, Ear2, Apoe, Ace, Ctsb, Eno3, Apoc2, Pou2f2, Cst3, Fabp4, Hfe, Cybb, Cd300e, Hpgd, Fcgr4, Klf4, Adgre4, Treml4, Ceacam1, Csf1r | [(7–10,15,16)](https://paperpile.com/c/mmaqtZ/UZbOW+Uqaw5+h5AZH+ALEoM+tiuCw+yfXCQ) |
|  | Bl.Macro.3 | **Monocyte-derived Foamy Plin2+ 2 (**Ly6c2, Lyz2, Cd68, Cx3cr1, Adgre1, Plin2, Cd36, Apoe, Ear2, Apoc2) | Chil3, Apoe, Lyz2, Ms4a6c, S100a4, Ear2, Apoc2, Plac8, Cybb, Ctss, Gngt2, Fn1, Ifi30, Ccr2, F13a1, Pou2f2, Ctsb, Cst3, Clec4a3, Crip1 | [(7–16)](https://paperpile.com/c/mmaqtZ/UZbOW+Uqaw5+h5AZH+ALEoM+tiuCw+yfXCQ+mOpo+r5WD+TDND+3yKn) |
| B Cells (Cd79a, Cd79b, H2-Aa, H2-Ab1) | Bl.B Cells.1 |  | Igkc, Cd79a, Cd74, Ly6d, Cd79b, H2-Aa, Iglc2, H2-Eb1, Iglc3, Iglc1, H2-Ab1, Ighm, Ebf1, Ms4a1, Fcmr, Ighd, H2-DMb2, Vpreb3, Mef2c, Mzb1 | [(6)](https://paperpile.com/c/mmaqtZ/PIih) |
|  | Bl.B Cells.2 |  | Ebf1, Gm31243, Mef2c, mt-Co3, BE692007, Ralgps2, mt-Nd2, mt-Atp6, Fchsd2, mt-Cytb, Bank1, mt-Co2, Ighm, mt-Nd1, Siglecg, mt-Nd3, Foxp1, mt-Nd4, Ighd, mt-Nd4l | [(6)](https://paperpile.com/c/mmaqtZ/PIih) |
|  | Bl.B Cells.3 |  | Igkc, Cd79a, Ly6d, Cd74, Ighm, H2-Eb1, H2-Aa, H2-Ab1, Cd79b, Ms4a1, Ebf1, Iglc3, Iglc2, Ighd, Ralgps2, Bank1, Gm31243, Fchsd2, Fcmr, Pax5 | [(6)](https://paperpile.com/c/mmaqtZ/PIih) |
| Hybrid B and T Cells (H2-Aa, H2-Ab1, Cd3d, Cd3e, Cd79a, Cd79b) | Bl.Hybrid B and T Cells | | Iglc1, Cd79a, Igkc, Cd74, Trbc2, Ly6d, H2-Eb1, Ighm, Iglc2, Cd79b, H2-Aa, Fcmr, Rpl12, Ighd, Rps20, H2-Ab1, Cd3d, Rps7, Rpl36a, Rps15a | [(6)](https://paperpile.com/c/mmaqtZ/PIih) |
| Natural Killer Cells (Trbc1, Trbc2, Klrk1, Klrg1, Klre1, Klrb1c, Klrb1b, Krlb1a) | Bl.NK.1 |  | Gzma, Ccl5, AW112010, Nkg7, Prf1, Gzmb, Klre1, Ncr1, Klra4, Ccl4, Klrb1c, Klrd1, Lgals1, Klrk1, Klra8, Klra9, Id2, Ctsw, Cma1, Xcl1 | [(6)](https://paperpile.com/c/mmaqtZ/PIih) |
|  | Bl.NK.2 |  | Stmn1, Hist1h1b, Hist1h2ae, Pclaf, Tuba1b, Top2a, Tubb5, Hist1h2ab, Hist1h3c, Lgals1, Hist1h4d, Hist1h1e, Dut, Ptma, Gzma, H2afz, Ran, Hist1h2ap, Mki67, Birc5. | [(6)](https://paperpile.com/c/mmaqtZ/PIih) |
| Plasmacytoid Dendritic Cells (H2-Aa, H2-Ab1, Bst2addfdf and pDC markers Tcf4, Irf8, and Bst2) | Bl.pDC.1 | **Antigen Presentation** (H2-DMb1, H2-Ab1, H2-Aa) | H2-DMb1, H2-Ab1, H2-Aa, Cd209a, H2-Eb1, Ifi30, Cst3, Cd74, Crip1, Tcf4, H2-DMa, Bst2, Irf8, Ckb, S100a4, Tmem176b, Cd7, Lgals1, Ccnd1, Gng10 | [(6,17,18)](https://paperpile.com/c/mmaqtZ/PIih+wd3h+IC1F) |
|  | Bl.pDC.2 | **Plasmacytoid** (Bst2, Siglech, Tcf4, Irf8) | Bst2, Siglech, Cox6a2, Klk1, Ctsl, Irf8, Rnase6, Ly6d, Tcf4, Ccnd1, Gm21762, Ly6a, Dnajc7, Ccr9, Upb1, Pltp, Lgals1, Smim5, Fdps, Cd209d | [(6,17,18)](https://paperpile.com/c/mmaqtZ/PIih+wd3h+IC1F) |
| Monocytes (High Ly6c2) | Bl.Monocytes |  | Fn1, Ahnak, F13a1, Ccr2, Lrp1, Zeb2, Vcan, mt-Co3, mt-Co2, Rassf4, mt-Co1, mt-Atp6, Psap, mt-Nd4, mt-Cytb, mt-Nd3, Cybb, mt-Nd1, mt-Nd2, C3 | [(6)](https://paperpile.com/c/mmaqtZ/PIih) |
| Mast Cells (Fcer1a) | Bl.Mast Cells |  | Mcpt8, Prss34, Ccl3, Cd63, Ccl9, Fcer1a, Ms4a2, Cd200r3, Cyp11a1, Cpa3, Gata2, Nedd4, Ccl6, Csrp3, Ccl4, Hgf, Ifitm1, Tbc1d4, MPC2, Lat2 | [(6)](https://paperpile.com/c/mmaqtZ/PIih) |
| Contaminating Red Blood Cells (Hbb-bs, Hba-a1, Hba-a2, Alas2, Bpgm, Fech, Slc25a37, Gypa, Car2, Epb41) | Contaminating RBCs |  | Hbb-bs, Hba-a1, Hba-a2, Hbb-bt, Alas2, Bpgm, Snca, Ube2l6, Mkrn1, Car2, Tent5c, Prxl2a, Fech, Isg20, Gypa, Slc25a39, 2-Mar, Slc25a37, Ube2o, Epb41 | [(6)](https://paperpile.com/c/mmaqtZ/PIih) |

**B. Brain**

| **Cell Type and Markers** | **Subtype** | **Potential Function (enriched genes that align with function)** | **Top 20 enriched genes in subtype** | **References** |
| --- | --- | --- | --- | --- |
| Microglia (Cx3cr1, Siglech, P2ry12, Tmem119) | Br.Micro.1 | **Homeostatic** (Cst3, Gpr34, Csfr1, Mef2c, Fcrls, Gpr34) | P2ry12, Cst3, Gpr34, Cx3cr1, Sparc, Tmem119, Hexb, Selplg, Siglech, Crybb1, Ltc4s, Pmepa1, Olfml3, Ecscr, Selenop, Serinc3, Arhgap5, Rnase4, Hpgd | [(19,20)](https://paperpile.com/c/mmaqtZ/2aong+8FQP5) |
|  | Br.Micro.2 | **Activated** (Fos, Egr1, Ccl4, Cd83, Ccl3, Egr1, Jun, Junb, Zfp36, Jund, Btg, Rhob, Fosb, Dups1, Ler2, Socs3, Nfkbia, Zfp36l1, Btg1, Ptma, Sgk1, Klf6) | Egr1, Jun, Rhob, Jund, Ccl4, Cst3, Klf2, Kctd12, P2ry12, Cx3cr1, Ier5, Btg2, Atf3, Ubc, Zfp36, Ccl3, Ier2, Gpr34, Cited2, Hpgd | [(19)](https://paperpile.com/c/mmaqtZ/2aong) |
|  | Br.Micro.3 | Activated 2 (Fosb, Malat1, Jun, Egr1, Egr1, Ier5, Smad7) | Fosb, Gm26532, Malat1, Jun, Dleu2, Egr1, Tmx4, Kctd12, Slc38a2, Tle4, Ppp1r10, Sat1, Ier5, Srgap2, Tra2b, Rhoh, Jund, Skil, Smad7 | [(19)](https://paperpile.com/c/mmaqtZ/2aong) |
|  | Br.Micro.4 | **Disease-Associated** (Mt-nd2, Mt-nd4, Mt-nd3, Mt-co1) | Mt-cytb, Mt-co3, Mt-nd2, Mt-nd4, Mt-nd1, Mt-atp6, Mt-co2, Mt-nd4l, Srgap2, Mt-nd5, Ac149090.1, Mt-nd3, Malat1, Mt-co1, Nav2, Ivns1abp, Mycbp2, Tanc2, E230029c05rik, Phf14 | [(19,21,22)](https://paperpile.com/c/mmaqtZ/OatjW+2aong+BF0xu) |
|  | Br.Micro.5 | **Complement** (C1qa, C1qb, C1qc, Ccl4, Ifit3) | Ccl12, Ly86, Ccl4, Ifit3, Lgals3bp, Ctsl, C1qc, C1qb, Cd9, Bst2, Syngr1, Ctsd, Cd81, Ifit2, C1qa, Cd63, Ccl3, Ldhb, Aif1, Olfml3 | [(23,24)](https://paperpile.com/c/mmaqtZ/igAIE+ibo2j) |
| Macrophage (Cd68, Lyz2, cd14, cx3cr1) | Br.Macro.1 | **Anti-inflammatory** (Mrc1, Stab1, Gas6, Clec12a, and Lilrb4a) | Mrc1, Pf4, Apoe, H2-eb1, H2-ab1, H2-aa, Cd74, Dab2, Ms4a7, F13a1, Stab1, Wwp1, Clec12a, Gas6, Gpr65, Myo5a, Ccl2, Selenop, Ccl12, Lilrb4a | [(25)](https://paperpile.com/c/mmaqtZ/Tqsci) |
|  | Br.Macro.2 | **Monocyte-derived Non-foamy Plin2+** (Lyz2, Plin2, Cd36, Plac8, Ly6c2) | Apoc2, Tgfb1, Apoe, Lgals3, Thbs1, Lyz2, Chil3, Ly6c2, Ms4a6c, Ms4a7, Ms4a4c, Cstb, Ccr2, Plac8, Ifitm3, Fn1, Lgals1, Vim, Ms4a6d, Isg15 (Plin2 is 21) | [(11–14,26)](https://paperpile.com/c/mmaqtZ/mLUc+mOpo+r5WD+TDND+3yKn) |
|  | Br.Macro.3 | **Monocyte-derived Macrophage** (Cd68, S100a4, S100a6, S100a10, Ly6c2, Plac8) | Lyz2, Chil3, Plac8, Crip1, Hp, S100a4, Ifitm6, S100a6, Tmsb10, Vcan, Vim, Ly6c2, Mgst1, Ahnak, S100a10, F13a1, Ccl6, Ifitm2, Ifitm3, Cybb | [(11–14,26)](https://paperpile.com/c/mmaqtZ/mLUc+mOpo+r5WD+TDND+3yKn) |
|  | Br.Macro.4 | **Monocyte-Derived Foamy Plin2+**  (Plin2, Cd36, Plac8, Ly6c2) | Cybb, Ear2, Itgal, Ace, Pglyrp1, Ceacam1, Gngt2, Fabp4, Itga4, Adgre4, Spn, Stap1, Nr4a1, Fcgr4, Flna, Cd300ld, Stk10, Cd300e, Gpx1, Napsa | [(7–16)](https://paperpile.com/c/mmaqtZ/UZbOW+Uqaw5+h5AZH+ALEoM+tiuCw+yfXCQ+mOpo+r5WD+3yKn+TDND) |
|  | Br.Macro.5 | **Resident Non-foamy Plin2+** (Plin2, Spp1, Hmox1, Ftl1, Fabp5, Cd63, Lgals1 +/Cd36-) | Spp1, Hmox1, Ftl1, Fabp5, Mt1, Cd63, Prdx1, Gapdh, Mif, Fth1, Ftl1-ps1, Pkm, Lgals1, Plin2, Capg, Gng5, Npl, Tmem256, Cd72 | [(7–10,15,16)](https://paperpile.com/c/mmaqtZ/UZbOW+Uqaw5+h5AZH+ALEoM+tiuCw+yfXCQ) |
| Dendritic Cells (H2-Aa, H2-Ab1, Bst2) | Br.DC.1 | **Interferon Signaling** (Fitm1, Ifitm2, Ifitm3, Cd74, H2-eb1, H2-ab1, H2-dmb1, Crip1, Tms10, Ifi30) | Ifitm1, H2-eb1, H2-aa, H2-ab1, Cd74, H2-dmb1, Ifitm2, Klrd1, Crip1, Ly6a, Napsa, Plbd1, S100a11, Ifitm3, Fxyd5, S100a4, Tmsb10, Ifi30, Aa467197, Ifi205 | [(27)](https://paperpile.com/c/mmaqtZ/WxGI3) |
|  | Br.Dc.2 | **Chemotaxis** (Ccr7, Ccl5) | Ccr7, Ccl5, Tbc1d4, Fabp5, Traf1, Tmem123, Crip1, Lsp1, Bcl2a1d, Relb, Il4i1, Fscn1, Zmynd15, Psme2, Calm1, Gadd45b, Marcksl1, H2afz, H2-eb1, Cacnb3 | [(27)](https://paperpile.com/c/mmaqtZ/WxGI3) |
|  | Br.DC.3 | **Plasmacytoid** (Ly6d) | Ly6d, Cox6a2, Rnase6, Ccr9, Ly6a, Bst2, Ly6c2, Irf8, Dnajc7, Bcl11a, Iglc3, Gm21762, Klk1, Atp1b1, Smim5, Runx2, Cd8b1, St8sia4, Rpl31, Rell1 | [(6)](https://paperpile.com/c/mmaqtZ/PIih) |
| Cd8+ T Cells (Cd3d, Cd3g, Cd3e, Cd8a, Cd8b1, Il7r, Trbc1, Trbc2) | Br.Cd8+ T Cell |  | Trbc2, Ccl5, Cd3g, Ms4a4b, Cd3d, Nkg7, Trbc1, Gimap3, Cd3e, Ltb, Il7r, Ctla2a, Cd8b1, Hcst, Trac, Bcl2, Aw112010, H2-q7, Gimap4, Tmsb10 | [(6)](https://paperpile.com/c/mmaqtZ/PIih) |
| Neutrophils (S100a8, S100a9, Cxcr2, Ly6g) | Br.Neut.1 | **Chemotaxis** (Cxcl2, Mmp9, Retnlg, Csf3r, Il1b, Tnfaip2, G0s2) | S100a8, S100a9, Cxcl2, Retnlg, G0s2, Il1b, Hdc, Mmp9, Cxcr2, Tnfaip2, Slpi, Slc7a11, Pim1, Clec4e, Lrg1, Csf3r, Ptgs2, Igfbp6, Resf1, Mxd1 | [(1,28)](https://paperpile.com/c/mmaqtZ/Izrpi+tVXb) |
|  | Br.Neut.2 | **Anti-microbial** (Camp, Ngp, Lcn2, Ltf) | Ngp, Camp, S100a9, S100a8, Lcn2, Ltf, Retnlg, Wfdc21, Pglyrp1, Mmp8, Ifitm6, Anxa1, Cd177, Mmp9, Hp, Syne1, G0s2, Chil3, Ly6g, Slpi | [(1,28)](https://paperpile.com/c/mmaqtZ/Izrpi+tVXb) |
| B Cells (Cd79a, Cd79b, H2-Aa, H2-Ab1) | Br.B Cells.1 |  | Igkc, Cd79a, Ly6d, Ebf1, Cd79b, Iglc2, Iglc1, Ighm, Iglc3, Vpreb3, Ms4a1, Ighd, Mzb1, Cd55, Be692007, Cd74, Fcmr, Ltb, Ptprcap, Chchd10 | [(6)](https://paperpile.com/c/mmaqtZ/PIih) |
|  | Br.B Cells.2 |  | Igkc, Cd79a, Ighm, Ebf1, Ly6d, Iglc2, Cd74, Cd79b, Apoc2, Iglc3, Iglc1, Ms4a1, H2-aa, Lyz2, Tgfb1, H2-ab1, Apoe, H2-eb1, Ighd, Chil3 | [(6)](https://paperpile.com/c/mmaqtZ/PIih) |
| Natural Killer Cells (Trbc1, Trbc2, Klrk1, Klrg1, Klre1, Klrb1c, Klrb1b, Krlb1a) | Br.NK |  | Gzma, Ccl5, Aw112010, Nkg7, Gzmb, Prf1, Ms4a4b, Klrb1c, Txk, Klre1, Klrk1, Il2rb, Klrd1, Ncr1, Xcl1, Klra9, Klra4, Bcl2, Ctsw, Ugcg | [(6)](https://paperpile.com/c/mmaqtZ/PIih) |
| Unknown Cell (high cell cycle, DNA replication, and mitosis replicator genes Stmn1, Top2a, Mki67, Cenpf, Bric5, Ube2c, Ube2s, Prc1, Cdca8, Histones) |  |  | Stmn1, Hist1h1b, Top2a, Hist1h2ap, Hist1h2ae, Birc5, Ube2c, Spp1, Pclaf, Mki67, Tubb5, Hmgb2, Tuba1b, Cenpf, H2afz, Prc1, Cdca8, Tubb4b, Ube2s, Selenoh | [(6)](https://paperpile.com/c/mmaqtZ/PIih) |
| Contaminating Astrocytes (Slc1a2, Aldoc, Atp1a2), Neurons (Camk2a, Camk2n1, Nrgn, Kif5a), and oligodendrocytes (Mbp) |  |  | Bc1, Slc1a2, Mt3, Aldoc, Camk2n1, Clu, Pcsk1n, Atp1a2, Cpe, Sparcl1, Ptn, Mbp, Gpm6b, Camk2a, Ndrg2, Kif5a, Scd2, Nrgn, mt-Nd2, mt-Nd4 | [(6)](https://paperpile.com/c/mmaqtZ/PIih) |
| Contaminating Endothelial Cells (Cldn5, Flt1, Egfl7, high Pecam1) |  |  | Ly6c1, Igfbp7, Flt1, Cldn5, Cxcl12, Bsg, Sptbn1, Ctla2a, Itm2a, Ptn, Spock2, Egfl7, Ramp2, Slco1a4, Ly6a, Hspb1, Sparcl1, Slc2a1, Tsc22d1, Pltp | [(6)](https://paperpile.com/c/mmaqtZ/PIih) |

**References**

1. [Xie X, Shi Q, Wu P, Zhang X, Kambara H, Su J, et al. Single-cell transcriptome profiling reveals neutrophil heterogeneity in homeostasis and infection. Nat Immunol. 2020 Sep;21(9):1119–33.](http://paperpile.com/b/mmaqtZ/tVXb)

2. [Hu T, Cheng B, Matsunaga A, Zhang T, Lu X, Fang H, et al. Single-cell analysis defines highly specific leukemia-induced neutrophils and links MMP8 expression to recruitment of tumor associated neutrophils during FGFR1 driven leukemogenesis. Exp Hematol Oncol. 2024 May 10;13(1):49.](http://paperpile.com/b/mmaqtZ/kd8du)

3. [McLaren AS, Fetit R, Wood CS, Falconer J, Steele CW. Single cell sequencing of neutrophils demonstrates phenotypic heterogeneity and functional plasticity in health, disease, and cancer. Chin Clin Oncol. 2023 Apr;12(2):18.](http://paperpile.com/b/mmaqtZ/QOehi)

4. [Castillo-Dela Cruz P, Wanek AG, Kumar P, An X, Elsegeiny W, Horne W, et al. Intestinal IL-17R signaling constrains IL-18-driven liver inflammation by the regulation of microbiome-derived products. Cell Rep. 2019 Nov 19;29(8):2270–83.e7.](http://paperpile.com/b/mmaqtZ/57RIU)

5. [Wang Y, Wan R, Hu C. Leptin/obR signaling exacerbates obesity-related neutrophilic airway inflammation through inflammatory M1 macrophages. Mol Med. 2023 Jul 24;29(1):100.](http://paperpile.com/b/mmaqtZ/1Y7Cw)

6. [CellMarker2.0 [Internet]. [cited 2025 Feb 7]. Available from:](http://paperpile.com/b/mmaqtZ/PIih) <http://www.bio-bigdata.center/>

7. [Gautam S, Banerjee M. The macrophage Ox-LDL receptor, CD36 and its association with type II diabetes mellitus. Mol Genet Metab. 2011 Apr;102(4):389–98.](http://paperpile.com/b/mmaqtZ/UZbOW)

8. [Collot-Teixeira S, Martin J, McDermott-Roe C, Poston R, McGregor JL. CD36 and macrophages in atherosclerosis. Cardiovasc Res. 2007 Aug 1;75(3):468–77.](http://paperpile.com/b/mmaqtZ/Uqaw5)

9. [Okamura DM, Pennathur S, Pasichnyk K, López-Guisa JM, Collins S, Febbraio M, et al. CD36 regulates oxidative stress and inflammation in hypercholesterolemic CKD. J Am Soc Nephrol. 2009 Mar;20(3):495–505.](http://paperpile.com/b/mmaqtZ/h5AZH)

10. [Loix M, Wouters E, Vanherle S, Dehairs J, McManaman JL, Kemps H, et al. Perilipin-2 limits remyelination by preventing lipid droplet degradation. Cell Mol Life Sci. 2022 Sep 13;79(10):515.](http://paperpile.com/b/mmaqtZ/ALEoM)

11. [Mysore V, Tahir S, Furuhashi K, Arora J, Rosetti F, Cullere X, et al. Monocytes transition to macrophages within the inflamed vasculature via monocyte CCR2 and endothelial TNFR2. J Exp Med [Internet]. 2022 May 2;219(5). Available from:](http://paperpile.com/b/mmaqtZ/mOpo) <http://dx.doi.org/10.1084/jem.20210562>

12. [Tacke F, Alvarez D, Kaplan TJ, Jakubzick C, Spanbroek R, Llodra J, et al. Monocyte subsets differentially employ CCR2, CCR5, and CX3CR1 to accumulate within atherosclerotic plaques. J Clin Invest. 2007 Jan;117(1):185–94.](http://paperpile.com/b/mmaqtZ/r5WD)

13. [Amengual J, Barrett TJ. Monocytes and macrophages in atherogenesis. Curr Opin Lipidol. 2019 Oct;30(5):401–8.](http://paperpile.com/b/mmaqtZ/TDND)

14. [Dash SP, Gupta S, Sarangi PP. Monocytes and macrophages: Origin, homing, differentiation, and functionality during inflammation. Heliyon. 2024 Apr 30;10(8):e29686.](http://paperpile.com/b/mmaqtZ/3yKn)

15. [Prentice KJ, Saksi J, Hotamisligil GS. Adipokine FABP4 integrates energy stores and counterregulatory metabolic responses. J Lipid Res. 2019 Apr;60(4):734–40.](http://paperpile.com/b/mmaqtZ/tiuCw)

16. [Furuhashi M, Saitoh S, Shimamoto K, Miura T. Fatty acid-binding protein 4 (FABP4): Pathophysiological insights and potent clinical biomarker of metabolic and cardiovascular diseases. Clin Med Insights Cardiol. 2014;8(Suppl 3):23–33.](http://paperpile.com/b/mmaqtZ/yfXCQ)

17. [Musumeci A, Lutz K, Winheim E, Krug AB. What makes a pDC: Recent advances in understanding plasmacytoid DC development and heterogeneity. Front Immunol. 2019 May 29;10:1222.](http://paperpile.com/b/mmaqtZ/wd3h)

18. [Valente M, Collinet N, Vu Manh TP, Popoff D, Rahmani K, Naciri K, et al. Novel mouse models based on intersectional genetics to identify and characterize plasmacytoid dendritic cells. Nat Immunol. 2023 Apr;24(4):714–28.](http://paperpile.com/b/mmaqtZ/IC1F)

19. [Pettas S, Karagianni K, Kanata E, Chatziefstathiou A, Christoudia N, Xanthopoulos K, et al. Profiling microglia through single-cell RNA sequencing over the course of development, aging, and disease. Cells. 2022 Aug 2;11(15):2383.](http://paperpile.com/b/mmaqtZ/2aong)

20. [Butovsky O, Weiner HL. Microglial signatures and their role in health and disease. Nat Rev Neurosci. 2018 Oct;19(10):622–35.](http://paperpile.com/b/mmaqtZ/8FQP5)

21. [Jurga AM, Paleczna M, Kuter KZ. Overview of general and discriminating markers of differential microglia phenotypes. Front Cell Neurosci. 2020 Aug 6;14:198.](http://paperpile.com/b/mmaqtZ/OatjW)

22. [Jiang CT, Wu WF, Deng YH, Ge JW. Modulators of microglia activation and polarization in ischemic stroke (Review). Mol Med Rep. 2020 May;21(5):2006–18.](http://paperpile.com/b/mmaqtZ/BF0xu)

23. [Cruz-Rivera YE, Perez-Morales J, Santiago YM, Gonzalez VM, Morales L, Cabrera-Rios M, et al. A selection of important genes and their correlated behavior in Alzheimer’s disease. J Alzheimers Dis. 2018;65(1):193–205.](http://paperpile.com/b/mmaqtZ/igAIE)

24. [Wang PL, Yim AKY, Kim K, Avey D, Czepielewski RS, Colonna M, et al. Peripheral nerve resident macrophages are microglia-like cells with tissue-specific programming [Internet]. Immunology. bioRxiv; 2019. Available from:](http://paperpile.com/b/mmaqtZ/ibo2j) <https://www.biorxiv.org/content/10.1101/2019.12.19.883546v1.full>

25. [Willemsen L, de Winther MP. Macrophage subsets in atherosclerosis as defined by single-cell technologies. J Pathol. 2020 Apr;250(5):705–14.](http://paperpile.com/b/mmaqtZ/Tqsci)

26. [Al-Rifai R, Vandestienne M, Lavillegrand JR, Mirault T, Cornebise J, Poisson J, et al. JAK2V617F mutation drives vascular resident macrophages toward a pathogenic phenotype and promotes dissecting aortic aneurysm. Nat Commun. 2022 Nov 3;13(1):6592.](http://paperpile.com/b/mmaqtZ/mLUc)

27. [Robbins SH, Walzer T, Dembélé D, Thibault C, Defays A, Bessou G, et al. Novel insights into the relationships between dendritic cell subsets in human and mouse revealed by genome-wide expression profiling. Genome Biol. 2008 Jan 24;9(1):R17.](http://paperpile.com/b/mmaqtZ/WxGI3)

28. [Chen S, Zhang Q, Lu L, Xu C, Li J, Zha J, et al. Heterogeneity of neutrophils in cancer: one size does not fit all. Cancer Biol Med. 2022 Dec 12;19(12):1629–48.](http://paperpile.com/b/mmaqtZ/Izrpi)
