# Supplementary figures and images for: Obesity supersizes macrophage and neutrophil activation after stroke while lipid droplets play a protective role
Source: J Neuroinflammation. 2026 Mar 19;23:158. doi: 10.1186/s12974-026-03774-7 (PMC13188555; doi:10.1186/s12974-026-03774-7)

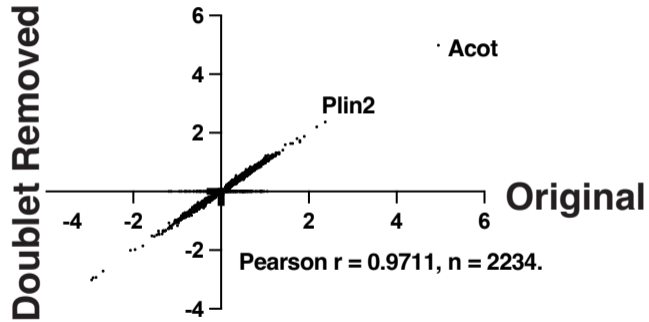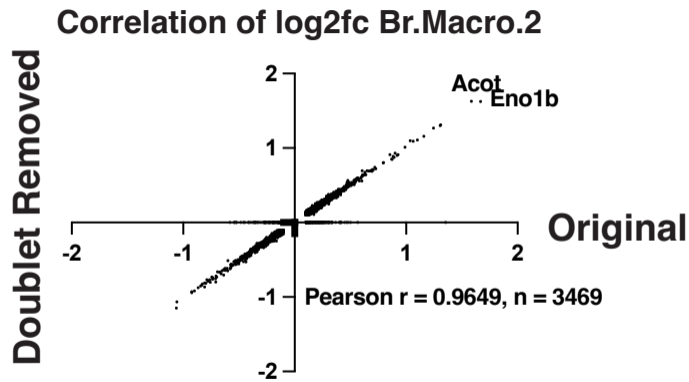

Supplement: Supplementary file 3 — Additional file 3. Supplementary Figure 1. Predicted doublets do not alter biological interpretations. Description: Correlation of log₂FC values for all differentially expressed genes (FDR < 0.05) in Bl.Neut.1 (left) and Br.Macro.2 (right) comparing analyses performed with and without predicted doublets identified by DoubletFinder. Pearson’s correlation coefficient for both immune cell subtypes exceeds 0.9, indicating high agreement. In both approaches, Eno1b and Acot1 remain among the most significantly affected genes, confirming that doublet handling does not meaningfully change the biological conclusions. [file 12974_2026_3774_MOESM3_ESM.pdf]

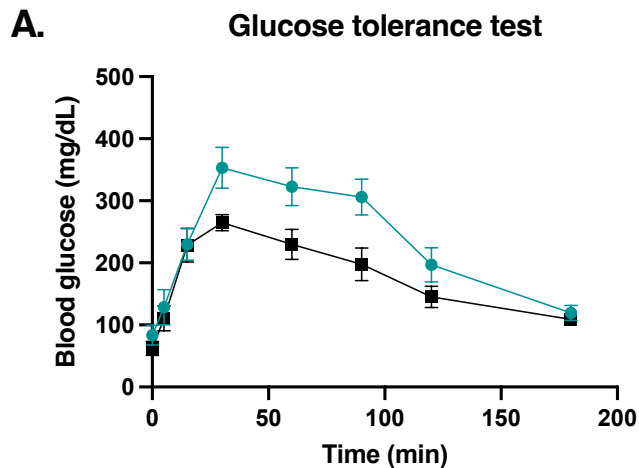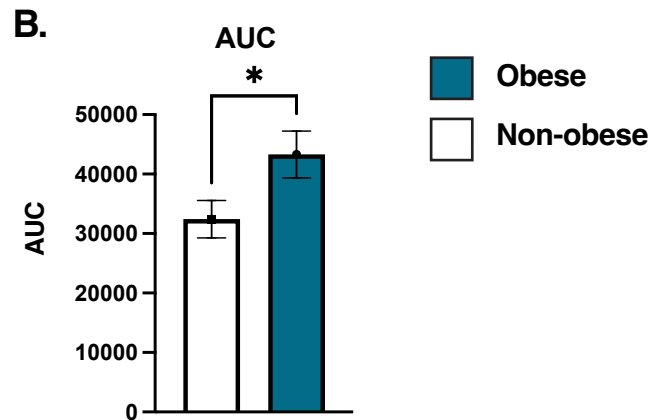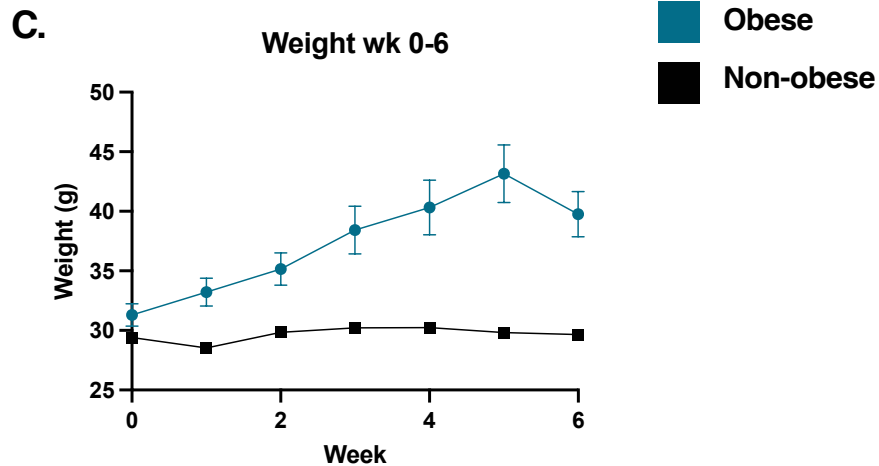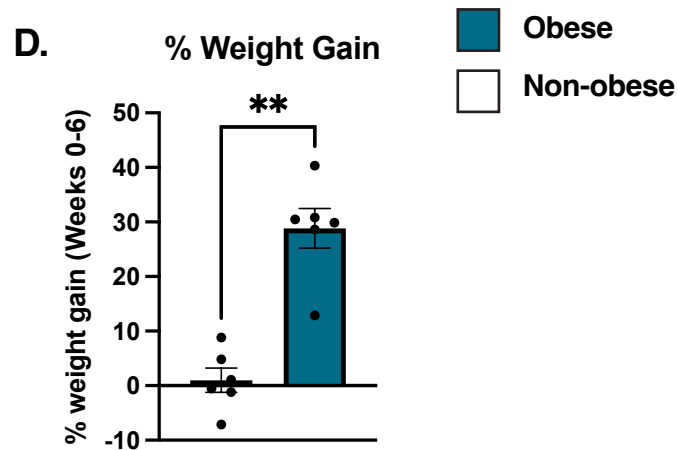

Supplement: Supplementary file 4 — Additional file 4. Supplementary Figure 2. Metabolic changes in response to a high fat diet. Description: (A-B) Curve and area under the curve for the glucose tolerance test. Welch’s T-test used to compare area under the curve (*p<0.05) (C-D) Weight over time and percent of weight gain between week 0 and week 6 of the high fat diet. Mann-Whitney test was used to compare percentage of weight gain (** p<0.001). [file 12974_2026_3774_MOESM4_ESM.pdf]

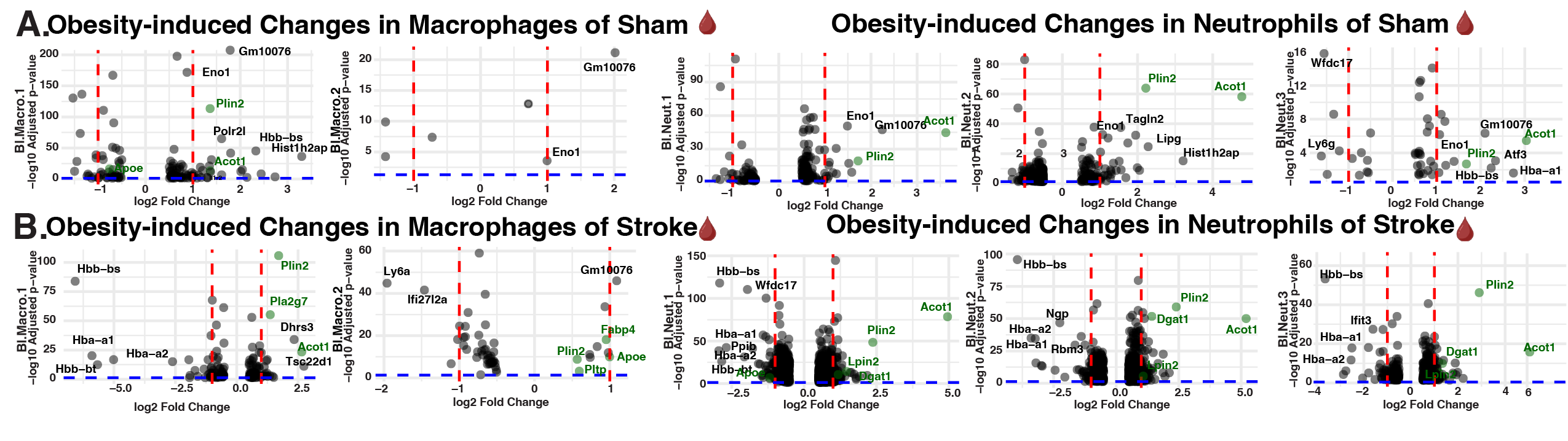

Supplement: Supplementary file 5 — Additional file 5. Supplementary Figure 3. Obesity-induced transcriptional changes in blood macrophages and neutrophils in mice after sham or stroke surgery. Description: Volcano plots showing differentially expressed genes induced by obesity in blood macrophages and neutrophils from mice after sham (A) and stroke (B) surgeries. Log2fc cut-off=|0.5| and fdr=0.05. Lipid-related genes are labeled in green. (C) MAP of blood and brain top affected macrophages. [file 12974_2026_3774_MOESM5_ESM.png]

# *Plin2* Expression After Stroke

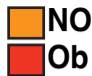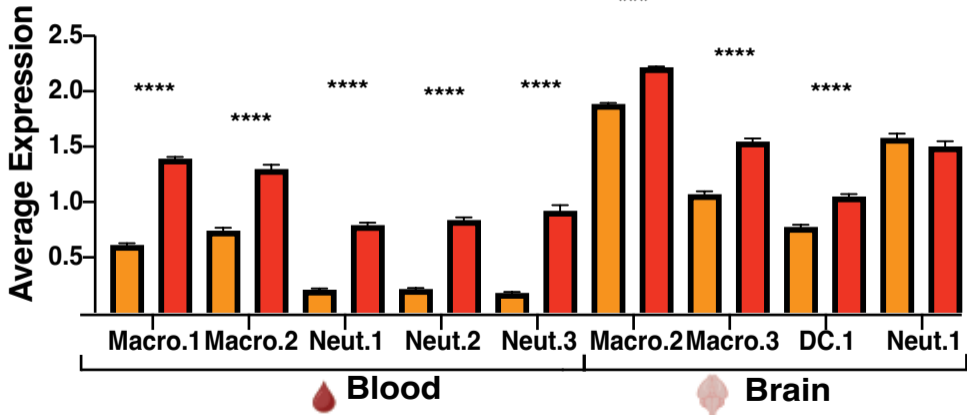

Supplement: Supplementary file 6 — Additional file 6. Title: Supplementary Figure 4. Average Plin2 gene expression after stroke. Description: Average gene expression of Plin2 in top changing blood and brain immune cells. ****- p<0.00001in MAST differential gene expression analysis. NO, non-obese; Ob, obese. [file 12974_2026_3774_MOESM6_ESM.pdf]

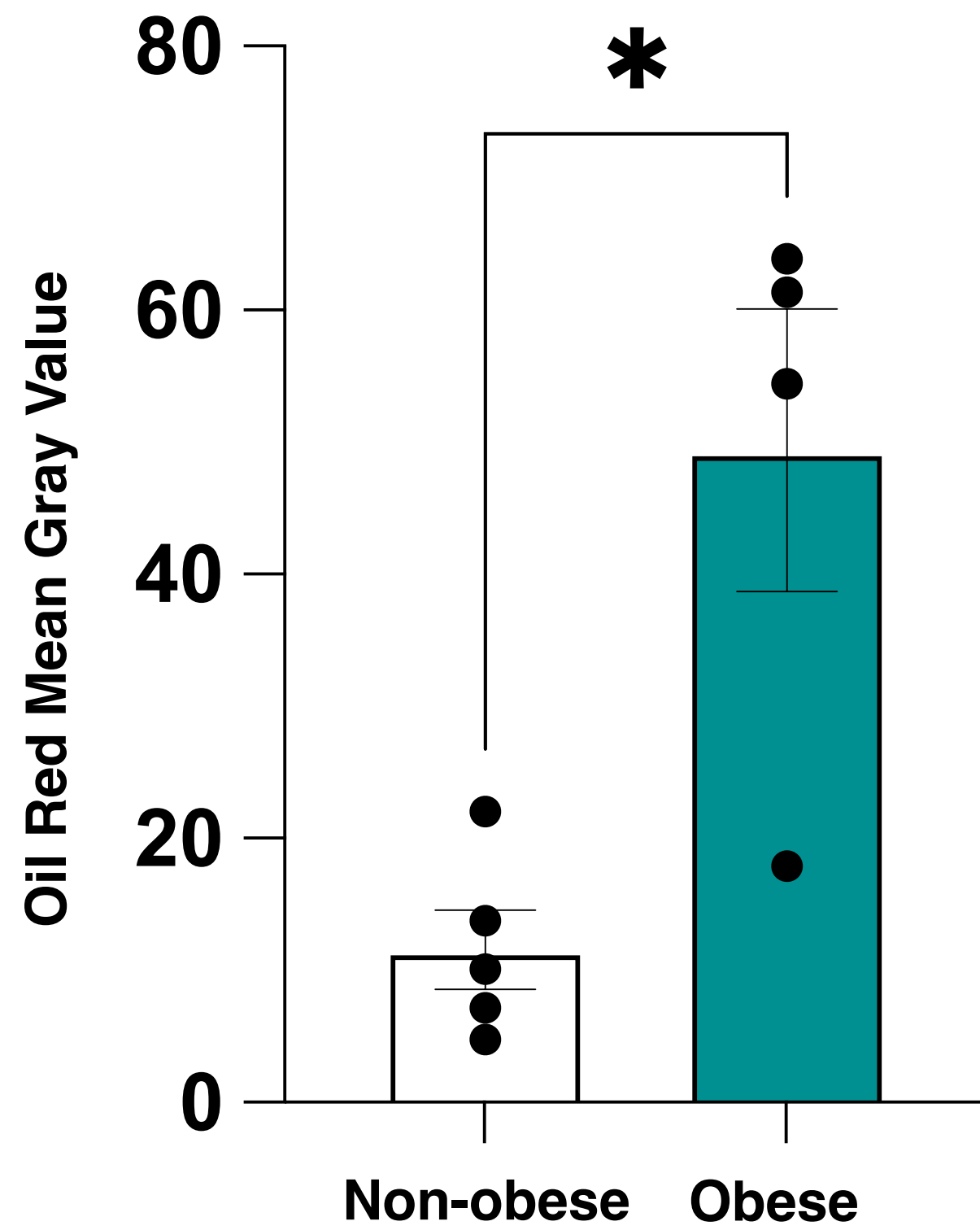

**NON-OBESE**

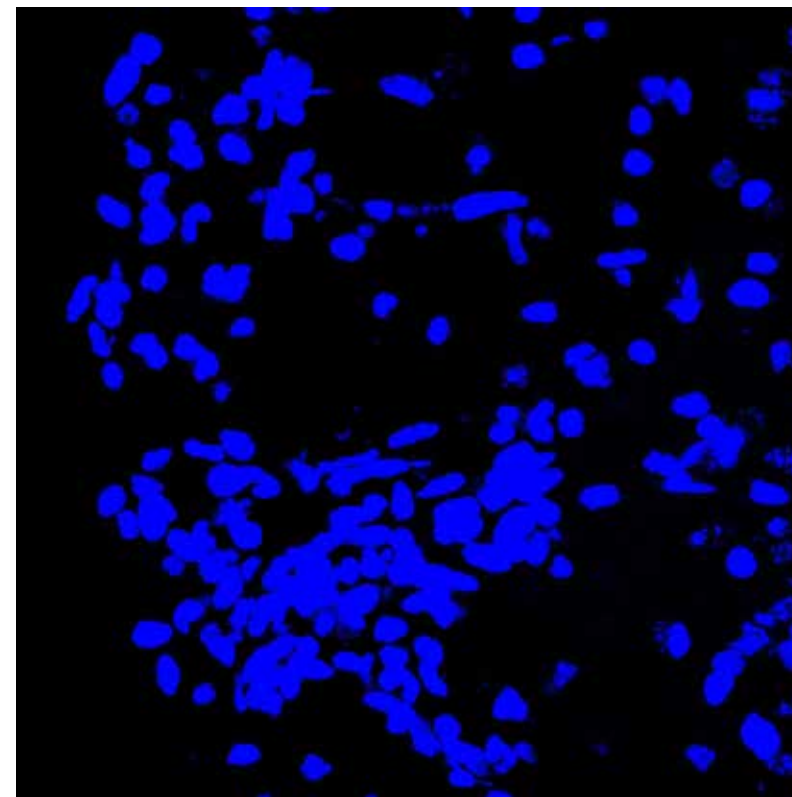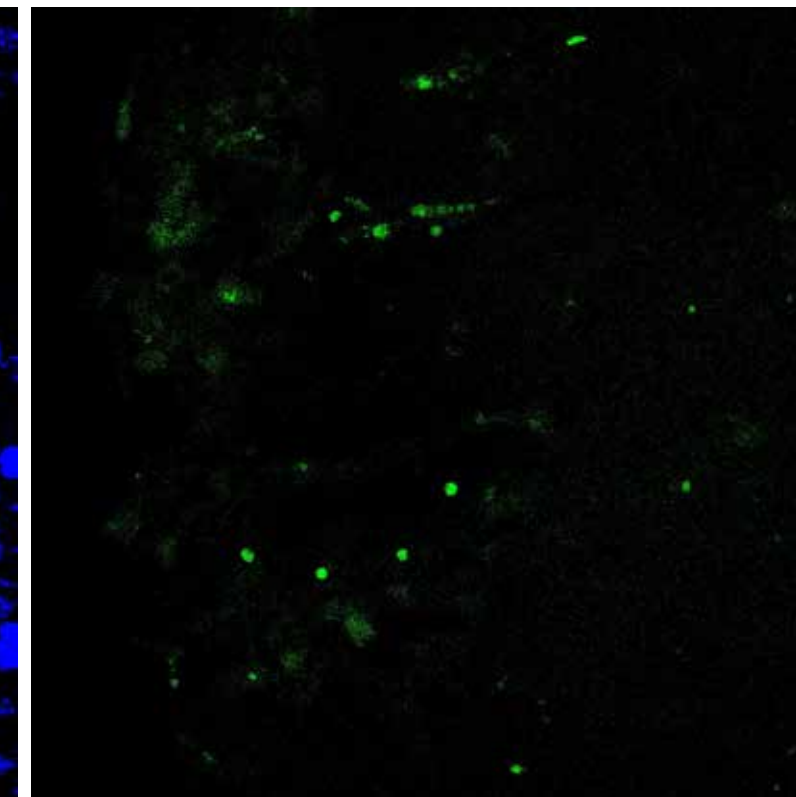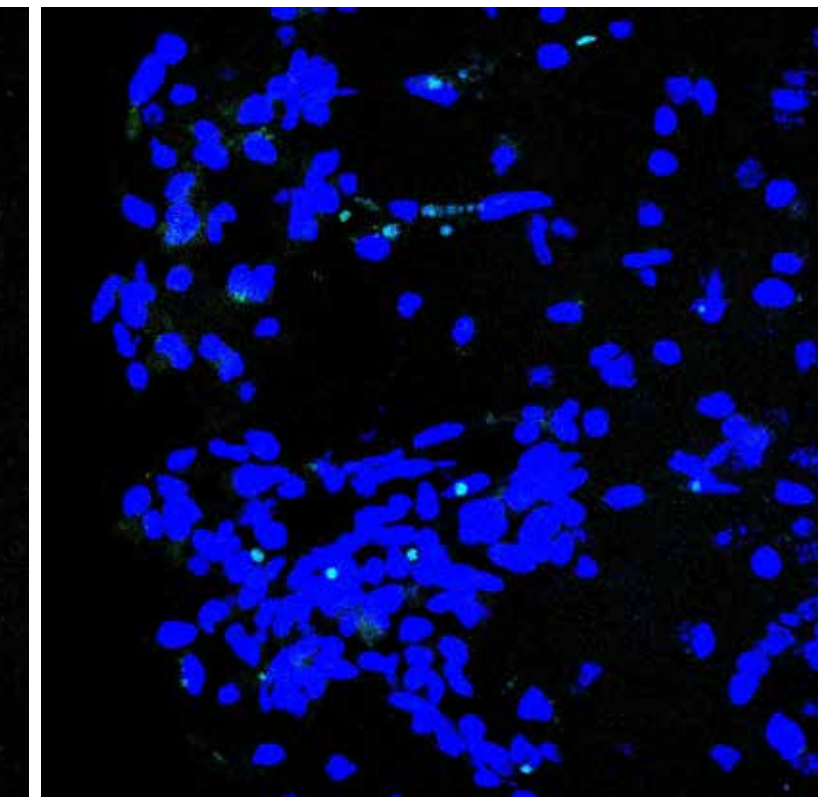

**OBESE**

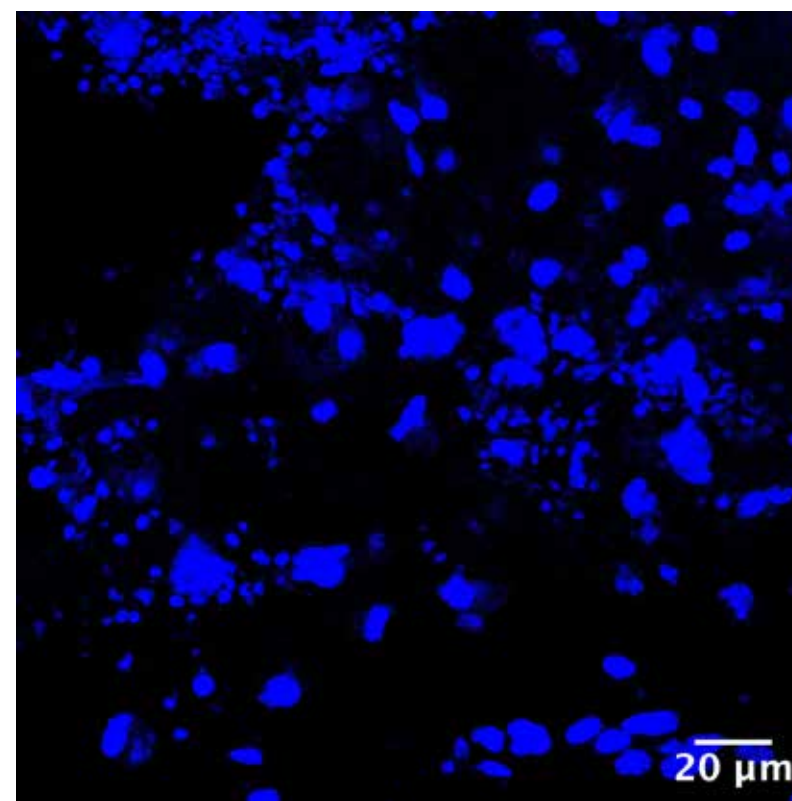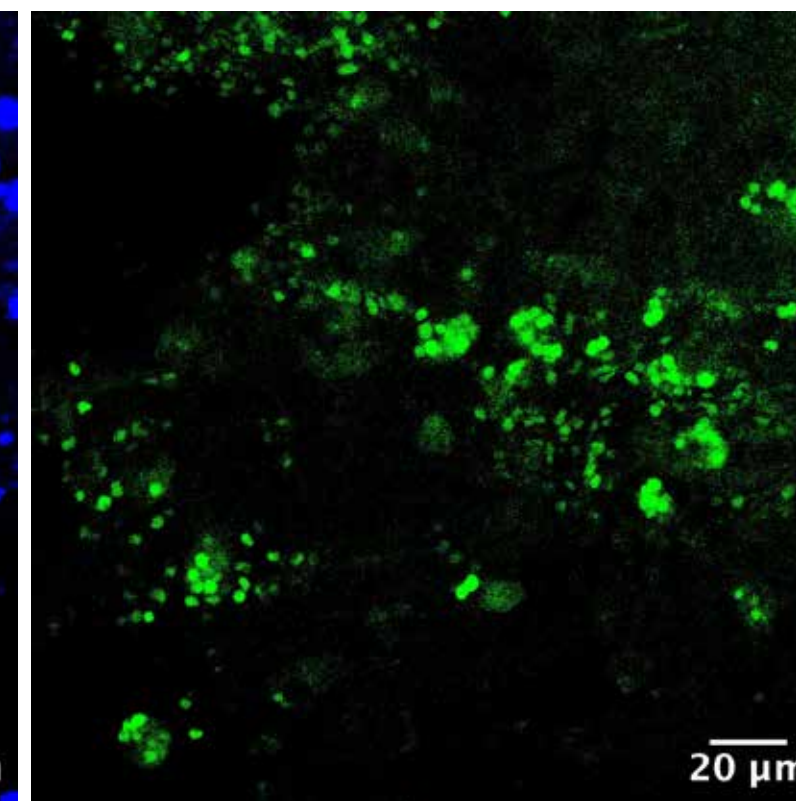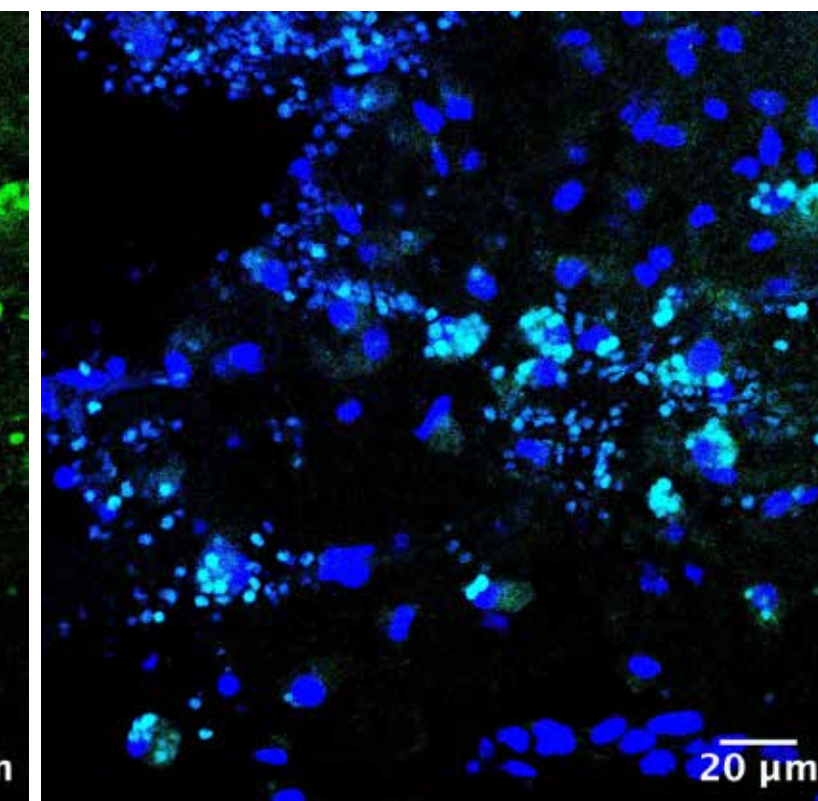

Supplement: Supplementary file 7 — Additional file 7. Supplementary Figure 5: Oil Red staining is elevated in obese mice compared to non-obese mice three days after stroke. Description: Quantification (left) and representative images (right) of oil red in stroke core (40X objective, n=3 sections/mouse, n=4-5 mice/group). Student t-test was used for statistical comparison (* p<0.05). [file 12974_2026_3774_MOESM7_ESM.pdf]

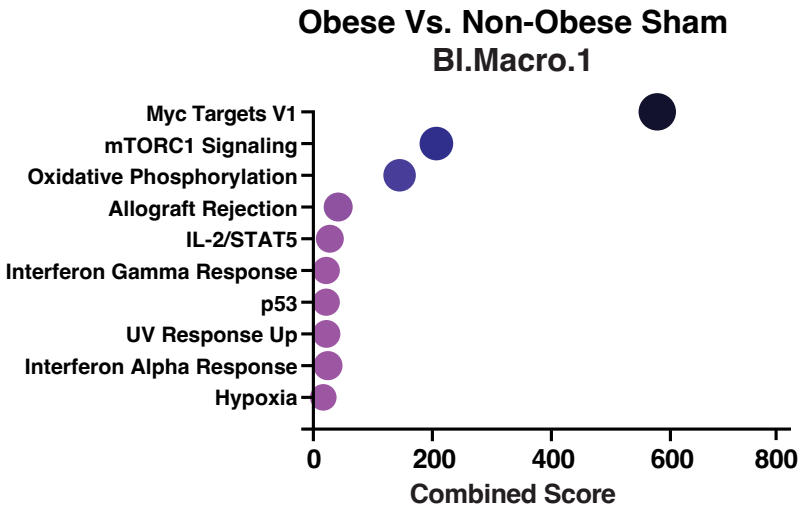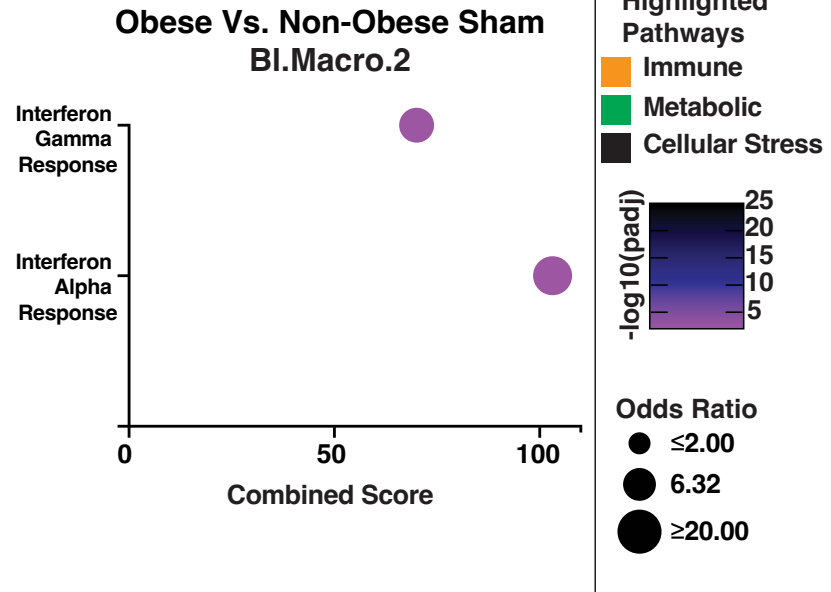

Supplement: Supplementary file 8 — Additional file 8. Supplementary Figure 6. Top obesity-enriched pathways in blood macrophages in mice after sham surgery. Description: Top obesity-changed pathways in Bl.Macro.1 (left) and Bl.Macro.2 (right) of mice after sham surgery. Pathways are from the MSigDB Hallmark 2020 database (fdr < 0.05). [file 12974_2026_3774_MOESM8_ESM.pdf]

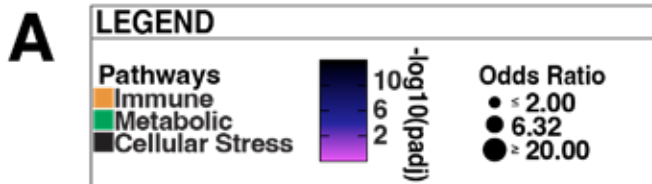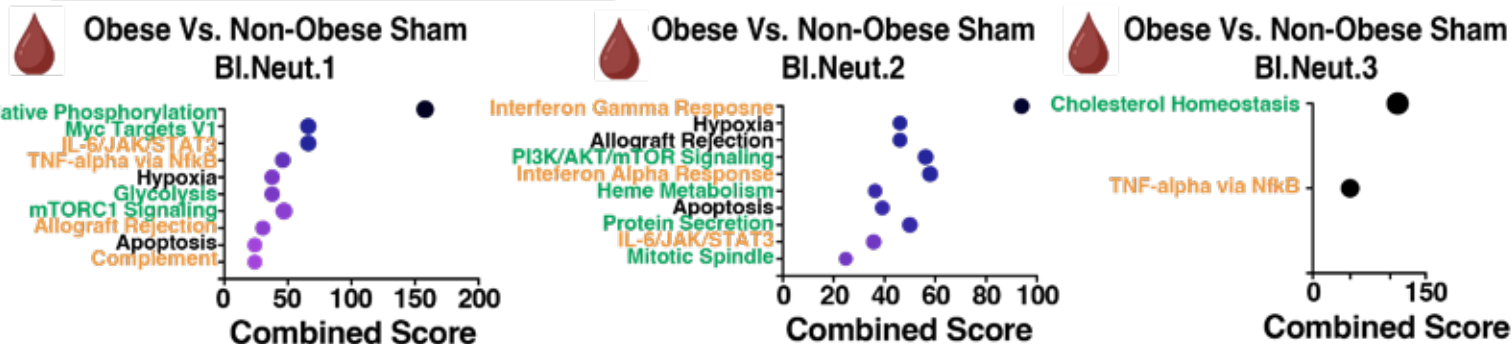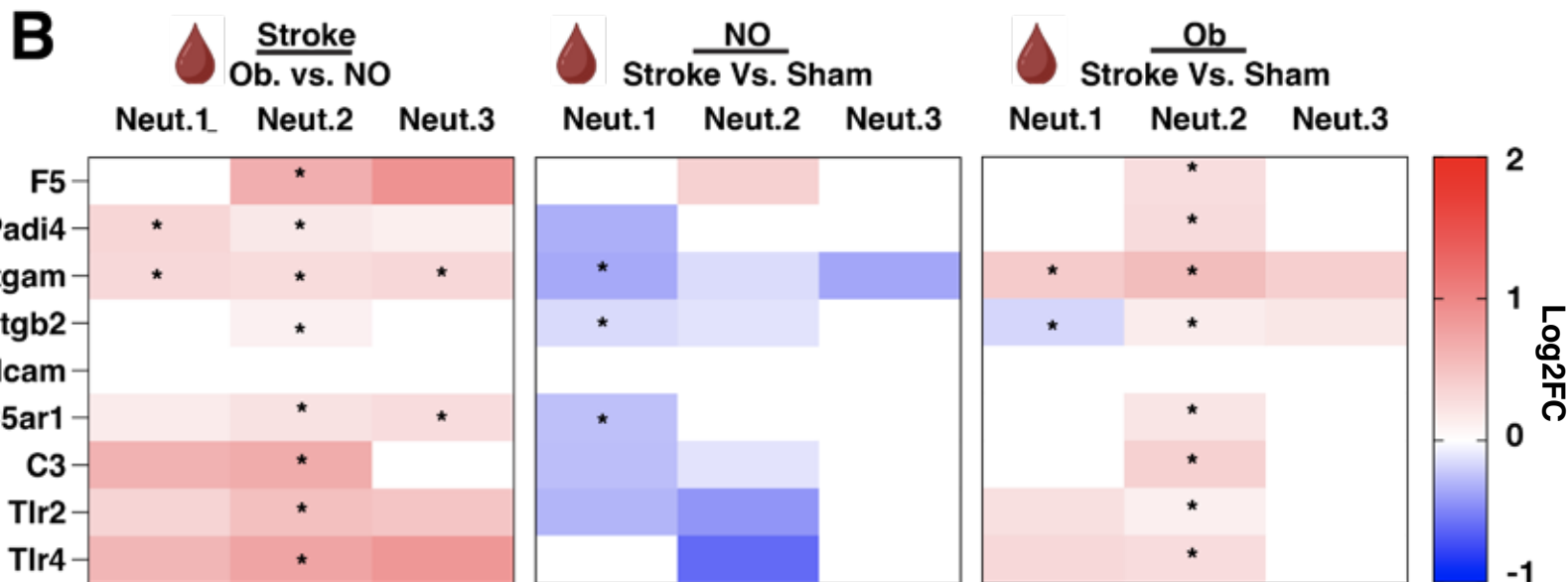

Supplement: Supplementary file 9 — Additional file 9. Supplementary Figure 7. Top obesity-enriched pathways and coagulation gene changes in blood neutrophils in mice after sham surgery. Description: Top obesity-changed pathways in Bl.Neut.1 (left), Bl.Neut.2 (middle), and Bl.Neut.3 (right) of mice after sham surgery. Pathways are from the MSigDB Hallmark 2020 database, genes used had fdr < 0.05. (B) Heatmap of classic coagulation genes changed by obesity after stroke in blood neutrophils. [file 12974_2026_3774_MOESM9_ESM.pdf]

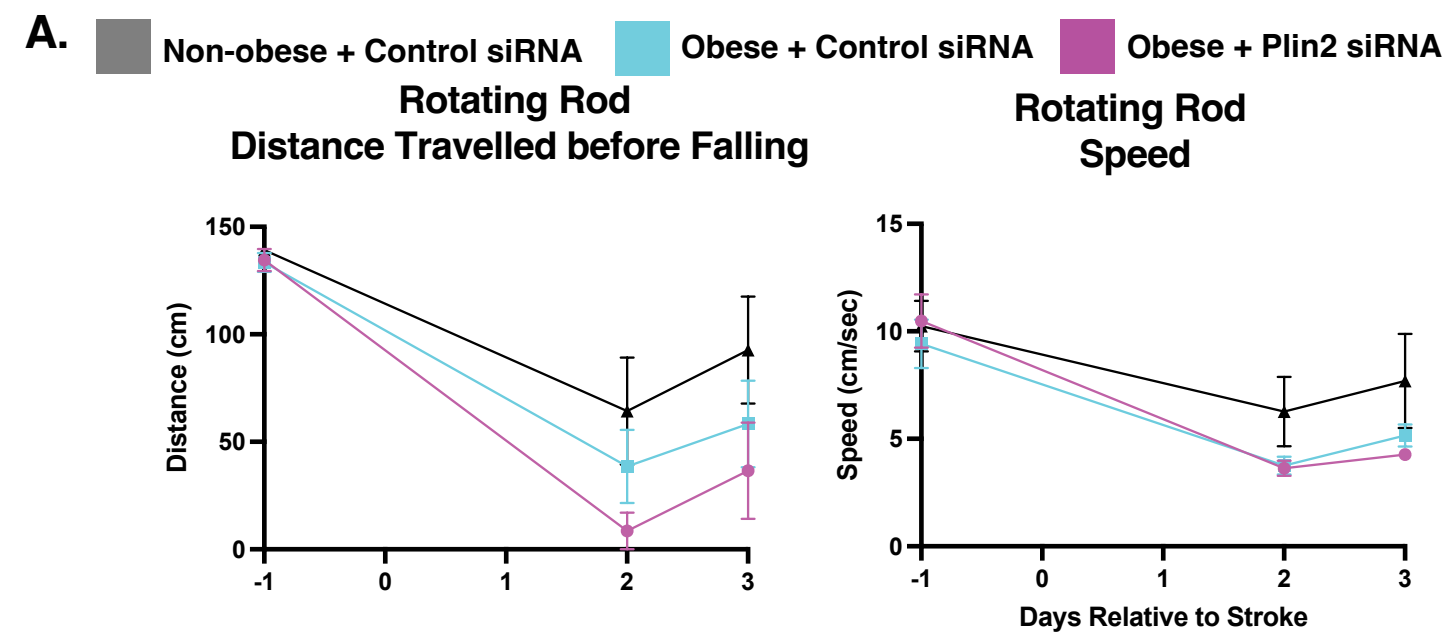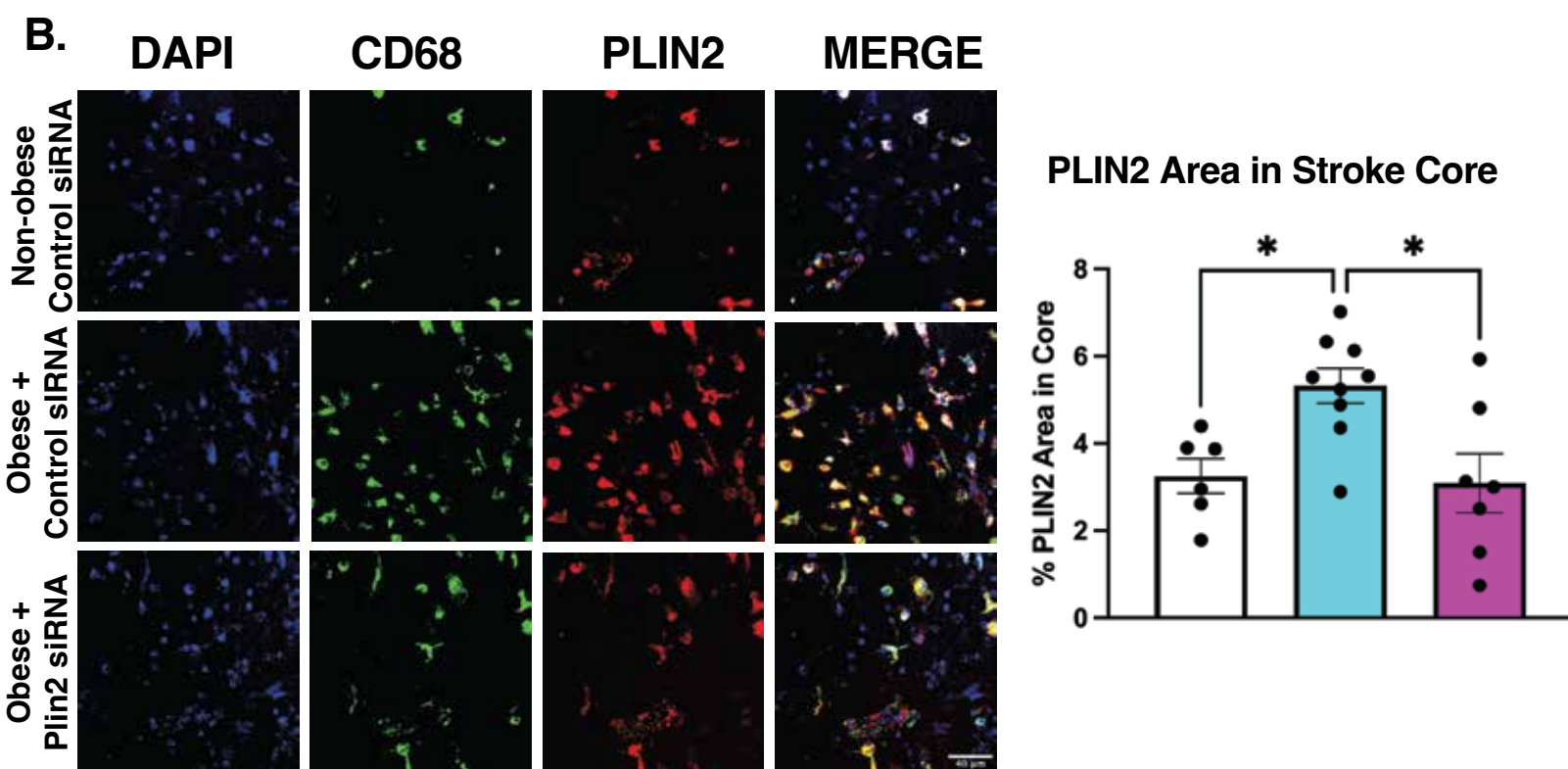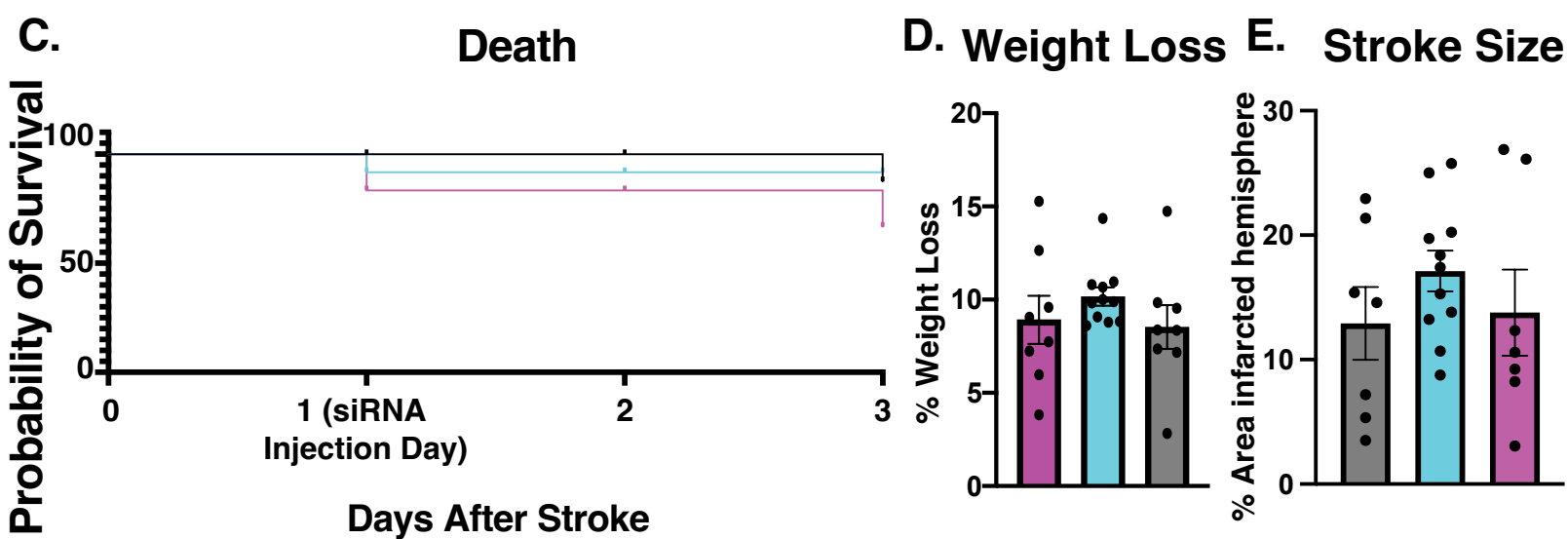

Supplement: Supplementary file 10 — Additional file 10. Supplementary Figure 8. Plin2 siRNA does not change weight loss or rotating rod performance in obese mice. Description: (A) Rotating Rod task performance defined by distance mice traveled before falling (left) and mouse speed (right). SEM bars are displayed. * p-value <0.05 in mixed model, Tukey’s post-hoc analysis (​​n=8 for non-obese mice, n=14-16 for obese mice). (B) Representative PLIN2 fluorescence image in the stroke core of obese mice (left) and quantification(right). (C) Death curve after stroke surgery (D) Weight loss of mice after stroke on day 3. (E) Quantification of stroke size established by lack of NEUN stain (n=7 sections, n=6-9 mice). Statistics, Student’s t-test; *p < 0.05; Bars, mean ± SEM. [file 12974_2026_3774_MOESM10_ESM.pdf]
